# An innovative Community Mobilisation and Community Incentivisation for child health in rural Pakistan (CoMIC): a cluster-randomised, controlled trial

**DOI:** 10.1016/S2214-109X(24)00428-5

**Published:** 2024-12-18

**Authors:** Jai K Das, Rehana A Salam, Zahra Ali Padhani, Arjumand Rizvi, Mushtaq Mirani, Muhammad Khan Jamali, Imran Ahmed Chauhadry, Imtiaz Sheikh, Sana Khatoon, Khan Muhammad, Rasool Bux, Anjum Naqvi, Fariha Shaheen, Rafey Ali, Sajid Muhammad, Simon Cousens, Zulfiqar A Bhutta

**Affiliations:** a Institute for Global Health and Development, Aga Khan University, Karachi, Pakistan; b Department of Paediatrics and Child Health, Aga Khan University, Karachi, Pakistan; c Centre of Excellence in Women and Child Health, Aga Khan University, Karachi, Pakistan; d The Daffodil Centre, The University of Sydney, a joint venture with Cancer Council NSW, Sydney, NSW, Australia; e Robinson Research Institute, Adelaide Medical School, University of Adelaide, Adelaide, SA, Australia; f London School of Hygiene & Tropical Medicine, London, UK; g Centre for Global Child Health, The Hospital for Sick Children, Toronto, ON, Canada

## Abstract

**Background:**

Infectious diseases remain the leading cause of death among children younger than 5 years due to disparities in access and acceptance of essential interventions. The Community Mobilisation and Community Incentivisation (CoMIC) trial was designed to evaluate a customised community mobilisation and incentivisation strategy for improving coverage of evidence-based interventions for child health in Pakistan.

**Methods:**

CoMIC was a three-arm cluster-randomised, controlled trial in rural areas of Pakistan. Clusters were formed by grouping villages based on geographical proximity, ethnic consistency, and ensuring a population between 1500 to 3000 per cluster. Clusters were randomly assigned (1:1:1) to either community mobilisation, community mobilisation and incentivisation, or the control arm. Community mobilisation included formation of village committees which conducted awareness activities, while clusters in the community mobilisation and incentivisation group were provided with a novel conditional, collective, community-based incentive (C3I) in addition to community mobilisation. C3I was conditioned on serial incremental targets for collective improvement in coverage at cluster level of three key indicators (primary outcomes): proportion of fully immunised children, use of oral rehydration solution, and sanitation index, assessed at 6 months, 15 months, and 24 months, and village committees decided on non-cash incentives for people in the villages. Data were analysed as intention-to-treat by an independent team masked to study groups. The trial is registered at ClinicalTrials.gov, NCT03594279, and is completed.

**Findings:**

Between Oct 1, 2018 and Oct 31, 2020, 21 638 children younger than 5 years from 24 846 households, with a total population of 139 005 in 48 clusters, were included in the study. 16 clusters comprising of 152 villages and 7361 children younger than 5 years were randomly assigned to the community mobilisation and incentivisation group; 16 clusters comprising of 166 villages and 7546 children younger than 5 years were randomly assigned to the community mobilisation group; and 16 clusters comprising of 139 villages and 6731 children younger than 5 years were randomly assigned to the control group. Endline analyses were conducted on 3812 children (1284 in the community mobilisation and incentivisation group, 1276 in the community mobilisation group, and 1252 in the control group). Multivariable analysis indicates improvements in all primary outcomes including a higher proportion of fully immunised children (risk ratio [RR] 1·3 [95% CI 1·0–1·5]), higher total sanitation index (mean difference 1·3 [95% CI 0·6–1·9]), and increased oral rehydration solution use (RR 1·5 [1·0–2·2]) in the community mobilisation and incentivisation group compared with the control group at 24 months. There was no evidence of difference between community mobilisation and control for any of the primary outcomes.

**Interpretation:**

Community mobilisation and incentivisation led to enhanced acceptance evidenced by improved community behaviours and increased coverage of essential interventions for child health. These findings have the potential to inform policy and future implementation of programmes targeting behaviour change but would need evaluation for varying outcomes and different contexts.

**Funding:**

Bill & Melinda Gates Foundation.

**Translations:**

For the Sindhi and Urdu translations of the abstract see Supplementary Materials section.

## Introduction

Global mortality in children younger than 5 years has reduced by more than half since 2000, with the annual number of deaths in this age group falling to 4·9 million from 9·9 million.^1^ Despite this, 13 400 children younger than 5 years died every day in 2022, largely due to preventable causes,^1^ and these deaths were disproportionately clustered in the poorest regions. In 2022, four in five deaths in children younger than 5 years occurred in sub–Saharan Africa and southern Asia, with southern Asia accounting for 26% of global deaths in this age group.^1^ Infectious diseases, including pneumonia, diarrhoea, and malaria, remain among the leading causes of deaths due to disparities in access and acceptance of basic health interventions.

**Research in context**

**Evidence before this study**

Diarrhoea and pneumonia remain the leading causes of post-neonatal deaths in children younger than 5 years, despite being preventable and treatable. Community mobilisation and community-based incentives have been evaluated previously showing modest and variable improvements in coverage of reproductive, maternal, and child health indicators. Incentives evaluated thus far have been ‘’individual” whether conditional, unconditional, vouchers, pay for performance, microcredit, food and nutrition subsidies, and social support or user fee reductions, and these have shown small and variable effects on child health. We searched MEDLINE via Ovid for studies using the Medical Subject Headings and key terms related to diarrhoea, pneumonia, incentive, community mobilisation and child from database inception to Sept 28, 2024 (diarrhoea OR diarrhea OR pneumonia OR “acute respiratory infection” AND “incentive*” OR “community mobile$ation” OR mobile$ation OR mobile$* AND child*). We did not apply any date or language restrictions. There were no trials evaluating the effect of conditional collective community-based incentives on child health. We found studies assessing the effect of either performance-based financial incentives or individual-level conditional or unconditional cash transfers or food vouchers for improving maternal, newborn, and child health. Hence this can be recognised as a novel strategy which was evaluated in this study.

**Added value of this study**

The Community Mobilisation and CommunityIncentivisation (CoMIC) trial is an evaluation of a unique approach of community engagement and demand creation through community mobilisation and collective incentivisation to improve the uptake of essential child health interventions. To our knowledge, this trial is the first to assess the effectiveness of conditional community-based incentives involving serial incremental targets for collective improvement in community behaviours: proportion of children fully vaccinated, use of oral rehydration solution, and sanitation index. The findings from this trial indicate that positive behaviour changes are achievable if the community has autonomy and is provided with tangible short-term incentives, leading to wider community acceptance, and important improvements in the uptake of interventions for prevention and management of two leading causes of morbidity and mortality in children younger than 5 years.

**Implications of all the available evidence**

The CoMIC trial provides important insights for designing context-specific, community-directed interventions for child health at a larger scale and has the potential to inform policy and future implementation of programmes targeting behaviour change. The trial provides rigorous and pragmatic evidence on the effectiveness of a distinctive community mobilisation and incentivisation strategy, generating evidence from a country with one of the highest childhood mortality rates attributable to diarrhoea and pneumonia. The findings from the CoMIC trial have the potential to inform future programmes targeting behaviour change guidelines not only in Pakistan but also in similar low-income and middle-income countries with a high burden of childhood morbidity and mortality.

Globally, only half of newborns are breastfed within 1 h of birth, and 44% are exclusively breastfed until age 6 months.^2^ In 2023, an estimated 14·5 million children did not receive any vaccination (zero-dose children).^3^ Three in ten people lack a facility with water and soap, and 462 million children attend schools with no hygiene facilities.^4^, ^5^ The COVID-19 pandemic further disrupted health services and access.^6^, ^7^, ^8^ Despite the existing primary health-care facilities and a well-developed community health worker programme, Pakistan is at risk of failing to achieve Sustainable Development Goal 3.^9^ According to the Pakistan Demographic and Health Survey 2017–18,^10^ despite improvements in coverage, exclusive breastfeeding rates are 48%; use of oral rehydration solution for diarrhoea is 37%; treatment of acute respiratory infections (ARI) with antibiotics is 46%; and the proportion of fully immunised children is 66%, with wide disparities among provinces, districts, and urban and rural populations.^10^

Inclusion of behaviour change components is widely recognised when designing and implementing public health interventions and uptake of health interventions is influenced by various personal, cognitive, economic, social, cultural, and structural factors that are often resistant to change.^11^ Community mobilisation, broadly defined as “interventions to encourage community individuals or groups to plan, carry out, and evaluate activities on a participatory and sustained basis to improve their health and other needs”^12^ have been evaluated in the domains of maternal and child health.^13^ Incentives including user fees reduction and conditional and unconditional cash transfers have been evaluated to improve care-seeking for child health.^14^ However, most of the behaviour change programmes focusing merely on improving knowledge have shown few effects on health. Behaviour change interventions should ensure active engagement, acceptance, and understanding of the motivation to change, and adapt context-specific strategies to facilitate change (bottom-up approach) for sustainable improvements.^15^ This could be especially useful to aid with multifaceted issues related to child health including vaccine hesitancy^16^ and health-care seeking and can potentially improve coverage of essential interventions such as exclusive breastfeeding; oral rehydration solution uptake; and water, sanitation, and hygiene interventions (WASH).^17^

The Community Mobilisation and Community Incentivisation (CoMIC) trial was designed to implement a customised community mobilisation and incentivisation strategy and assess its effectiveness in improving uptake of evidence-based interventions and care-seeking practices for child health, especially for diarrhoea and pneumonia in rural Pakistan. The CoMIC trial utilised a previously untested strategy that did not involve conventional incentives at the individual level; rather, it proposed incremental non-cash conditional incentives at a collective, community level to foster behaviour change and broader community wellbeing.^18^, ^19^ The trial design for CoMIC was grounded in the Theory of Reasoned Action and the Theory of Planned Behaviour which focuses on how beliefs, subjective norms, attitudes towards the behaviour, and perceived behavioural control could lead to a behaviour intention to improve common childhood illnesses at the community level (appendix 3 p 1).^20^, ^21^

## Methods

### Study design and participants

CoMIC was a prospective, cluster-randomised, controlled trial, preceded by a formative phase, of which findings have been published previously.^19^ Briefly, the formative phase involved geo-spatial mapping of the study area to inform formation of clusters, focus group discussions and in-depth interviews with key stakeholders, and a baseline household survey. Findings from the formative phase assisted in identifying the existing practices along with context-specific barriers and facilitators for forming educational materials for improving child health behaviours.

**Figure 1:**
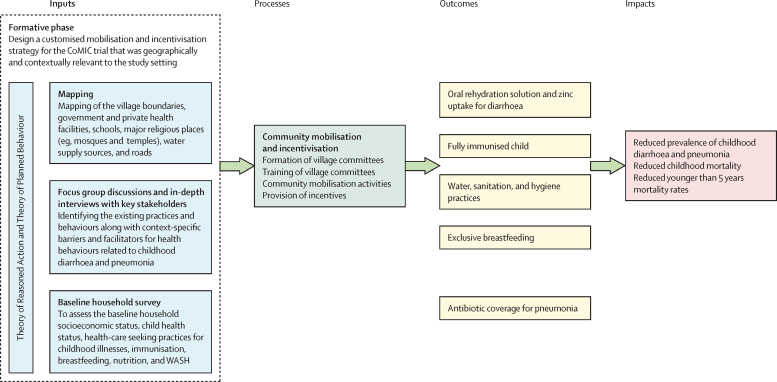
Logic pathway for CoMIC trial Adapted from Das and colleagues.^18^ CoMIC=Community Mobilisation and Community Incentivisation.

CoMIC was a three-arm randomised controlled trial with 16 clusters in each of the two interventions and a control group designed to assess the effect of community mobilisation alone and community mobilisation together with a conditional, collective, community-based incentive (C3I), on uptake and adherence to recommended preventive and therapeutic health interventions among children younger than 5 years in a rural setting of Pakistan. A cluster comprised a group of villages based on geographical proximity with a population of 1500–3000 individuals. The trial protocol, describing in detail the design, statistical analysis, and the CoMIC intervention, has been published previously (figure 1).^18^

The trial was conducted in the rural areas of Tando Muhammad Khan district in the province of Sindh, Pakistan. Tando Muhammad Khan has an area of 1814 km^2^ and a population of approximately 680 000 residents, administratively comprising of 17 Union Councils (lowest administrative unit).^10^ The intervention was delivered at the cluster level, while the target population for the evaluation was households with at least one child younger than age 5 years at the time of the survey, who was a permanent resident of the selected cluster.

The protocol was approved by the Aga Khan University Ethical Review Committee (2018–0349–470) and the National Bioethics Committee (4-87/17/NBC-224). The community was informed about the purpose, methods, benefits, and intended uses of research during the planning and approval meetings. Written informed consent and individual participant consent for data collection were obtained. The trial was prospectively registered with ClinicalTrials.gov with the registration number, NCT03594279. No safety monitoring board was set up as no potential adverse events and harms were anticipated from the community mobilisation activities.

### Randomisation and masking

Geo-spatial mapping was done and a total of 457 villages in ten Union Councils were selected to form study clusters by grouping villages based on geographical proximity (there was at least a 10 km distance between clusters), cultural or ethnic consistency within each cluster, and a population between 1500 and 3000. Randomisation was done by an independent statistician using a computer-generated randomisation sequence, and a total of 48 clusters were randomly assigned (1:1:1) to either the community mobilisation, community mobilisation and incentivisation, or control group through a covariate-constrained randomisation approach to balance clusters across socioeconomic status, education, and with at least 20 km distance between individual clusters. Covariate-constrained randomisation also minimised any potential biases arising from baseline imbalances, thereby enhancing the internal validity of our findings (appendix 3 pp 2–3). Participants could not be masked due to the nature of the interventions; however, data collection and analysis was done by independent teams which were not part of the core CoMIC team and were masked to the study groups.

### Procedures

Details of the interventions have previously been described separately^18^ and a condensed description is provided here. The overview of trial activities and timelines are summarised in appendix 3 (p 4).

The first intervention arm of community mobilisation involved awareness and mobilisation activities, and findings from the formative phase guided the development of educational materials and video messages. The villagers, through consensus, formed separate male and female village committees of six to eight members in each cluster. The study team conducted a 6-day training for these village committee members on specific messages in line with the study objectives of improving uptake of interventions including WASH, breastfeeding, nutrition, immunisation, and care-seeking practices for childhood diarrhoea and pneumonia. The training involved various modalities including education sessions, promotional videos, posters and flip charts, and group discussions with role play. Village committees were then responsible for carrying out community mobilisation activities in every village and school in their catchment area; every month for the first 6 months, every 2 months for the next 6 months, and quarterly in the second year. Community mobilisation activities included distribution of educational materials (posters, pictorial brochures, flip charts, and promotional videos in local language), community meetings, and health and awareness sessions in communities and schools (panel). The attendance of the committee members and villagers at the community meetings was recorded in registers along with geo-tagged pictures to check compliance.

Panel **Details of community mobilisation activities**

**Formation of village committees**

The village committees comprised of between six and eight members and included Union Council members, local elders and elites, religious leaders, and prominent male and female members of the community. Elites refers to influential people in the community and community elders are individuals who are recognised in their communities for their wisdom, and local cultural knowledge.

**Information education material**

These materials were prepared and distributed during the community sessions, and focused on the themes of water, sanitation and hygiene (WASH), breastfeeding, nutrition, immunisation, childhood diarrhoea and acute respiratory infection. These included posters, pictorial brochures and flip charts, and promotional videos in local (Sindhi) language.

**Meetings in the community**

The community awareness activities were held for males and females separately and involved village community meetings (triggering sessions), meetings supervised by research staff (participatory learning), and sessions with children in schools and the community (change leaders).

**Sessions in schools and communities for children**

Sessions conducted for children in schools and communities included educational sessions with key WASH and health-related messages; displays of information, education and communication materials inside school areas and classrooms; schoolteachers checking personal hygiene—clothes, nails, shoes, and hair; and competitions and games related to hygiene and nutrition including poster competitions and singing activities.

The second intervention arm of the trial was the community mobilisation and incentivisation group. In addition to the activities of the community mobilisation group, clusters in the community mobilisation and incentivisation group were provided with C3I, a novel incentive strategy focused on the broader health and wellbeing of the community. It aimed to encourage the community to work together to qualify for incentives based on serial incremental targets for improved indicators related to childhood illnesses of diarrhoea and pneumonia. To establish eligibility for C3I, a composite measure was devised based on the three primary outcomes of the CoMIC trial; proportion of fully immunised children, oral rehydration solution use, and sanitation index,^22^ each contributing an equal weight, and the community was expected to improve the coverage according to the following criteria. At 6 months: a 10% relative improvement in the composite coverage from baseline (a minimum of 5% improvement in each indicator); at 15 months: a 25% relative improvement (a minimum of 15% in each indicator); and at 24 months: a 50% relative improvement (minimum 30% in each indicator).

The village committees in the community mobilisation and incentivisation group were informed that they could qualify for three rounds of incentives if they improved their collective practices, but were not informed of the specific indicators. They were informed that the incentives would be non-cash and clusters that qualified at each round would be designated a fixed amount of money and these village committees would deliberate on what they would spend this on through a formal needs assessment and prioritisation exercise (appendix 3 p 5). The common incentives included water and sanitation facilities (as determined in the formative phase) and these incentives were then delivered by the study team and the total cost was shared by the project (75%) and the community (25%) to improve ownership and sustainability (appendix 3 p 6).

Clusters in the standard care control arm continued to receive routine child health care and sanitation management through existing structures and facilities.

At the baseline and endline, separate line-listing was carried out which served as a sampling frame for the selection of households with children younger than 5 years in respective surveys. A two-staged sampling technique was used, the sample size was equally divided between clusters in each group, and households were selected using the computer-generated randomised list. Independent separate field teams masked to the study groups conducted serial surveys (at 6 months, 15 months, and 24 months) to assess incentive eligibility and endline assessments. The content of the questionnaire and the complete data collection form are available in appendix 3 (pp 8, 14–44). The household information, including the demographic and socioeconomic measures, and child health outcome were obtained from the mother or caregiver of the child younger than 5 years after obtaining informed consent. Demographic and socioeconomic data included child's sex (caregiver reported), household ethnicity, religion, parents’ level of education, occupation, number of family members, ownership of the house, number of rooms used for sleeping, household construction materials, toilet facilities, sources of drinking water, household assets, and land ownership. The demographic health survey methods were used to categorise households into five wealth quintiles based on the possession of observable or easily asked about assets, services, and amenities. Data were collected using handheld devices (Samsung tablets running Android 5.1) after 6 days of training on content, operational procedures, and management. Range and consistency checks and skip patterns were built into the data entry program to minimise errors. Data were synced daily and uploaded from the field sites to the university server. The data management unit generated daily summary reports for quality check and, if required, sent the reports to the field teams for rectification. All the data were encrypted, secured, and fully anonymised.^18^

### Outcomes

Given that the objective of the CoMIC trial was to improve the uptake of evidence-based interventions for child health, in particular diarrhoea and pneumonia, the three prespecified primary outcomes were: the proportion of fully immunised children, defined as age-appropriate vaccination status for children up to 23 months of age based on vaccination cards or mother recall; oral rehydration solution use, defined as the proportion of children younger than 5 years who used oral rehydration solution for the last episode of diarrhoea; and sanitation index—adapted from Webb and colleagues.^22^ Sanitation index included three indicators: drinking water index, which comprised of interior water container water is covered, exterior water container is clean, and if the container contains water; food index, comprised of clean dishes are covered, clean dishes are stored high and all food is covered; and domestic household hygiene index, comprised of absence of trash (inside or outside the house), no unrestrained animal in house, no accumulation of dirty clothes, insignificant number of flies in house, and no standing water around the house. The three indicators (drinking water index, food index, domestic household hygiene index) comprised of a total of 12 items and each item was scored as 0 or 1, with 1 representing a positive behaviour. Sanitation index was calculated as the simple sum of all the items leading to a maximum score of 12 and was assessed with two spot checks at each assessment performed by different independent observers from the study team (appendix 3 p 7).

Secondary outcomes included exclusive breastfeeding rates (defined as no other food or drink, except breastmilk for 6 months of life); diarrhoea episode (passage of three or more loose or liquid stools per day within 2 weeks of the day of survey), and suspected acute respiratory infection prevalence (cough or difficult breathing, or both, with or without fever, within 2 weeks of the day of survey), care-seeking for childhood diarrhoea and pneumonia (professional help sought from health-care services, health-care providers, or community health workers), and open defecation rates (defecating in fields, bushes, or ditches).

### Statistical analysis

Findings from the baseline survey were used to calculate the required sample size based on each of the primary outcomes. In the baseline survey, oral rehydration solution use was 47·6%; fully immunised children coverage was 20·4%; while mean sanitation index was 5·63 (SD 1·7). Assuming a 50% relative improvement in the primary outcomes over the 2-year period, an intra-cluster coefficient of 0·05, an attrition of 10%, one-sided alpha level of 0·025 to account for two tests (for comparing community mobilisation *vs* control and community mobilisation and incentivisation *vs* control), a sample size of 1392 children per group was needed to provide 80% power. The maximum sample size calculated was 13 clusters in each group, but we included a total of 48 clusters with 16 clusters in each group to provide sufficient power and be prepared for any unforeseen problems during the trial.

The statistical analysis plan is specified in detail in the protocol.^18^ We summarised the categorical variables using frequencies and percentages; for continuous variables, we checked the distribution and, if approximately normally distributed, we summarised as means and SDs and if not, we summarised as medians and IQRs. We used the intention-to-treat population and assessed the effectiveness for each of the outcomes using a generalised linear model with robust standard errors to account for the effect of cluster randomisation. The binomial distribution was used with the log link function to estimate risk ratios (RR) and 95% CIs for binary outcomes such as the proportion of fully immunised children and oral rehydration use, and mean difference with SD for continuous outcomes such as sanitation index. For missing data on variables, which was very rare, we did not impute but treated this as missing and performed complete case analysis. We used the Gaussian distribution for continuous outcomes such as sanitation index. The estimates were adjusted for child sex, age, number of siblings, mother's education, father's education, mother's occupation, father's occupation, lady health worker visit, household density, and wealth quintiles for multivariable analysis. A generalised linear model was fitted through “binreg” and “glm” routines in STATA software, version 17.

We also conducted difference-in-differences analysis to analyse the association of the intervention with the outcomes by assembling the cross-sectional surveys.^23^ We used interaction estimators in unadjusted and covariate-adjusted regression methods to estimate the difference-in-differences effect. To calculate the degree of within-cluster dependence, the intraclass correlation coefficients were calculated by analysis of variance.

### Role of the funding source

The funders of the study had no role in study design, data collection, data analysis, data interpretation, or writing of the report.

## Results

**Figure 2:**
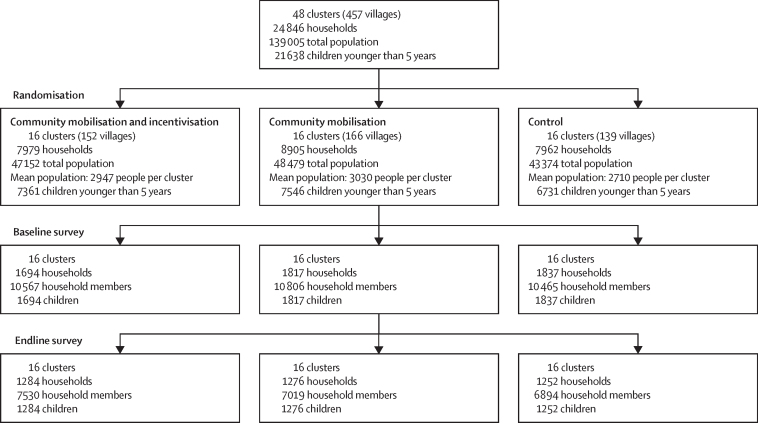
Trial profile The baseline survey was done before the intervention delivery while the endline survey was conducted at 24 months.

The study was conducted between Oct 1, 2018, and Oct 31, 2020 with a total of 21 638 children younger than 5 years from 24 846 households, with a total population of 139 005 in the 48 clusters at baseline. A total of 16 clusters comprising of 152 villages and 7361 children younger than 5 years were randomly assigned to the community mobilisation and incentivisation group; 16 clusters comprising of 166 villages and 7546 children younger than 5 years were randomly assigned to the community mobilisation group; while 16 clusters comprising of 139 villages and 6731 children younger than 5 years were randomly assigned to the control group (figure 2). The serial evaluations were done on a sub-sample based on the sample size calculated, separate line-listing was carried out to provide a sampling frame for the selection of households with children younger than 5 years for baseline and endline surveys. For baseline survey, assessment was conducted on 1694 children from the community mobilisation and incentivisation group; 1817 children from the community mobilisation group and 1837 children from the control group. The baseline characteristics and outcomes were similar across study arms (Table 1, Table 2); half of the mothers were literate, while literacy rate among fathers was 64% (2224 of 3462). Most of the mothers were not in paid employment, while most fathers were either skilled or unskilled workers. Nearly, one-third of the households reported use of improved sanitation (1763 [33·0%] of 5348) and almost a quarter (1172 [21·9%] of 5348) practised open defecation. The prevalence of diarrhoea was 26·2% (1356 of 5348) and acute respiratory infection was 23·4% (1248 of 5348), oral rehydration use for diarrhoea was reported for less than half of children (552 [40·6%] of 1356), 23·5% (862 of 3520) were exclusively breastfed for 6 months, and 28·2% (1093 of 3645) were fully immunised.

**Table 1:** Baseline characteristics

			**Total**	**Community mobilisation and incentivisation group**	**Community mobilisation group**	**Control group**
Household characteristics						
	Household completed		5348	1694	1817	1837
	Household density (number of people living in household)		5·5 (1·2)	5·6 (2·3)	5·5 (2·2)	5·3 (2·1)
Household wealth status						
	Poorest		997/5348 (18·6%)	277/1694 (16·4%)	385/1817 (21·2%)	335/1837 (18·2%)
	Poor		1040/5348 (19·4%)	323/1694 (19·1%)	344/1817 (18·9%)	373/1837 (20·3%)
	Middle		1091/5348 (20·4%)	362/1694 (21·4%)	382/1817 (21·0%)	347/1837 (18·9%)
	Rich		1158/5348 (21·7%)	389/1694 (23·0%)	407/1817 (22·4%)	362/1837 (19·7%)
	Richest		1062/5348 (19·9%)	343/1694 (20·2%)	299/1817 (16·5%)	420/1837 (22·9%)
Use of improved source of drinking water			5310/5348 (99·3%)	1689/1694 (99·7%)	1814/1817 (99·8%)	1807/1837 (98·4%)
Use of improved sanitation facilities			1763/5348 (32·9%)	603/1694 (35·6%)	563/1817 (30·9%)	597/1837 (32·5%)
Open defecation			1172/5348 (21·9%)	361/1694 (21·3%)	433/1817 (23·8%)	378/1837 (20·6%)
Main source of water for cooking and washing						
	Piped water		707/5348 (13·2%)	173/1694 (10·2%)	174/1817 (9·6%)	360/1837 (19·6%)
	Underground water		4611/5348 (86·2%)	1518/1694 (89·6%)	1642/1817 (90·4%)	1451/1837 (79%)
	Dug well		30/5348 (0·6%)	3/1694 (0·2%)	1/1817 (0·1%)	26/1837 (1·4%)
Place of water source						
	In own dwelling		3214/5348 (60·1%)	1084/1694 (64·0%)	1150/1817 (63·3%)	980/1837 (53·3%)
	In own yard or plot		1237/5348 (23·1%)	351/1694 (20·7%)	416/1817 (22·9%)	470/1837 (25·6%)
	Elsewhere		897/5348 (16·8%)	259/1694 (15·3%)	251/1817 (13·8%)	387/1837 (21·1%)
Household has electricity			3338/5348 (62·4%)	1100/1694 (64·9%)	1102/1817 (60·6%)	1136/1837 (61·8%)
Mean monthly income of household (PKR)			10 399 (35 182)	10 518 (19 957)	9492 (7648)	11 154 (55 414)
Spoken language of household						
	Sindhi		2589/5348 (48·4%)	759/1694 (44·8%)	924/1817 (50·9%)	906/1837 (49·3%)
	Dhadki		756/5348 (14·1%)	200/1694 (11·8%)	309/1817 (17·0%)	247/1837 (13·4%)
	Siraiki		1322/5348 (24·7%)	528/1694 (31·2%)	270/1817 (14·9%)	524/1837 (28·5%)
	Other		681/5348 (12·7%)	207/1694 (12·2%)	314/1817 (17·3%)	160/1837 (8·7%)
Lady health worker visiting the home			5329/5348 (99·6%)	1690/1694 (99·8%)	1811/1817 (99·7%)	1828/1837 (99·5%)
Parental and child characteristics						
	Maternal age, years		28·22 (6·24)	28·82 (6·19)	28·42 (6·38)	27·54 (6·09)
	Paternal age, years		32·23 (7·13)	32·75 (7·20)	32·18 (7·16)	31·83 (7·03)
	Mother's literacy*		1814/3505 (51·8%)	502/1097 (45·8%)	640/1135 (56·4%)	672/1273 (52·8%)
	Father's literacy*		2224/3462 (64·2%)	700/1082 (64·7%)	732/1123 (65·2%)	792/1257 (63%)
	Mother's employment*		396/3518 (11·3%)	126/1099 (11·5%)	168/1140 (14·7%)	102/1279 (8·0%)
	Father's occupation*					
		Unemployed	78/3462 (2·3%)	29/1082 (2·7%)	28/1123 (2·5%)	21/1257 (1·7%)
		Unskilled	1658/3462 (47·9%)	549/1082 (50·7%)	508/1123 (45·2%)	601/1257 (47·8%)
		Skilled	1512/3462 (43·7%)	432/1082 (39·9%)	523/1123 (46·6%)	557/1257 (44·3%)
		Professional or managerial	214/3462 (6·2%)	72/1082 (6·7%)	64/1123 (5·7%)	78/1257 (6·2%)
Total number of children younger than 5 years†			9471	3053	3138	3280
Age distribution of children younger than 5 years						
	0–2 years		3654/9471 (38·5%)	1149/3053 (37·6%)	1177/3138 (37·5%)	1325/3280 (40·4%)
	2–5 years		5820/9471 (61·5%)	1904/3053 (62·4%)	1961/3138 (62·5%)	1955/3280 (59·6%)
Sex						
	Male		4814/9471 (50·8%)	1538/3053 (50·4%)	1603/3138 (51·1%)	1673/3280 (51·0%)
	Female		4657/9471 (49·2%)	1515/3053 (49·6%)	1535/3138 (48·9%)	1607/3280 (49·0%)
	M:F ratio		1·03	1·02	1·04	1·04

Data are n/N (%) or mean (SD), unless otherwise specified. PKR=Pakistani rupee.

* Education and employment status were not reported for all parents.

† All present children younger than 5 years from each household were included in the analysis.

**Table 2:** Primary and secondary outcomes at baseline and endline

			**Total**	**Community mobilisation and incentivisation group**	**Community mobilisation group**	**Control group**
**Baseline**						
Outcomes at household level						
	Number of households with children younger than 5 years		5348	1694	1817	1837
	Sanitation index		6·9 (5·2)	6·7 (4·0)	6·9 (6·3)	6·9 (6·4)
		Drinking water index (score 0–3)	2·1 (2·4)	2·0 (2·4)	2·1 (2·8)	2·1 (2·9)
		Food index (score 0–3)	1·2 (2·8)	1·1 (2·5)	1·2 (3·0)	1·3 (3·2)
		Domestic household hygiene index (score 0–6)	3·6 (2·2)	3·5 (2·1)	3·7 (2·5)	3·6 (2·8)
	Open defecation		1172/5348 (22·1%)	361/1694 (21·3%)	433/1817 (23·8%)	378/1837 (20·6%)
Outcomes at child level						
	Number of children younger than 5 years		5348	1694	1817	1837
	Children with diarrhoea during past 2 weeks		1356/5348 (26·2%)	426/1694 (25·1%)	467/1817 (25·7%)	463/1837 (25·2%)
	Care-seeking for childhood diarrhoea		1097/1356 (81·7%)	338/426 (79·3%)	366/467 (78·4%)	393/463 (84·9%)
	Oral rehydration use for childhood diarrhoea		552/1356 (40·6%)	164/426 (38·5%)	181/467 (38·7%)	207/463 (44·7%)
	Children with acute respiratory infection during past 2 weeks		1248/5348 (23·4%)	404/1694 (23·9%)	421/1817 (23·2%)	423/1837 (23·0%)
	Antibiotic treatment for childhood acute respiratory infection		532/1248 (43·5%)	157/404 (38·8%)	180/421 (42·8%)	195/423 (46·1%)
	Number of children younger than 2 years		3645	1147	1173	1325
	Exclusive breastfeeding at 6 months		862/3520 (23·5%)	244/1100 (22·2%)	249/1140 (21·8%)	369/1280 (28·8%)
	Age-appropriate vaccination					
		Fully immunised	1093/3645 (28·2%)	359/1147 (31·3%)	341/1173 (28·9%)	393/1325 (29·5%)
		Partially immunised	1878/3645 (51·7%)	566/1147 (49·8%)	602/1173 (51·6%)	710/1325 (54·3%)
		Not immunised	674/3645 (20·2%)	222/1147 (19·2%)	230/1173 (19·4%)	222/1325 (16·1%)
**Endline**						
Outcomes at a household level						
	Number of households with children younger than 5 years		3812	1284	1276	1252
	Sanitation index		9·5 (8·6)	10·1 (7·5)	9·3 (7·2)	8·9 (7·4)
		Drinking water index (score 0–3)	2·4 (3·1)	2·7 (2·9)	2·4 (2·7)	2·3 (2·2)
		Food index (score 0–3)	2·5 (4·2)	2·9 (1·2)	2·3 (3·6)	2·2 (4·0)
		Domestic household hygiene index (score 0–6)	4·6 (3·3)	4·6 (3·7)	4·6 (2·8)	4·5 (3·3)
	Open defecation		1328/3812 (34·8%)	422/1284 (32·8%)	482/1276 (37·8%)	424/1252 (33·9%)
Outcomes at child level						
	Number of children younger than 5 years		3812	1284	1276	1252
	Children with diarrhoea during past 2 weeks		356/3812 (9·3%)	90/1284 (7·0%)	132/1276 (10·3%)	134/1252 (10·7%)
	Care-seeking for childhood diarrhoea		271/356 (76·1%)	69/90 (76·7%)	92/132 (69·7%)	110/134 (82·1%)
	Oral rehydration solution use for childhood diarrhoea		145/356 (40·7%)	48/90 (53·3%)	50/132 (37·9%)	47/134 (35·1%)
	Children with acute respiratory infection during past 2 weeks		168/3812 (4·4%)	51/1284 (4·0%)	54/1276 (4·2%)	63/1252 (5·0%)
	Antibiotic treatment for childhood acute respiratory infection		94/168 (56·0%)	31/51 (60·7%)	27/54 (50%)	36/63 (57·1%)
	Number of children younger than 2 years		1957	657	678	622
	Exclusive breastfeeding at 6 months		804/1866 (43·1%)	333/622 (53·5%)	256/647 (39·6%)	215/597 (36·0%)
	Age-appropriate vaccination					
		Fully immunised	968/1957 (49·8%)	380/657 (59·2%)	294/678 (43·7%)	294/622 (46·6%)
		Partially immunised	739/1957 (37·6%)	218/657 (32·3%)	283/678 (41·6%)	238/622 (38·9%)
		Not immunised	250/1957 (12·6%)	59/657 (8·5%)	101/678 (14·7%)	90/622 (14·6%)

Data are presented as n/N (%) or mean (SD). The estimates are adjusted to account for the clustering effect.

The total attendance in all the sessions over the 2-year period was 35 635 individuals in the community mobilisation and incentivisation group and 30 317 in the community mobilisation group. Based on interim surveys conducted at 6 months and 15 months in the 16 clusters of the community mobilisation and incentivisation group, 11 clusters met the required composite target at 6 months (10% improvement), while all 16 clusters met the desired target in the second interim survey at 15 months (25% improvement) and the endline survey at 24 months (50% improvement) and hence received the incentives decided according to the need prioritisation exercises (appendix 3 p 9). A total of 514 incentives were given (mostly water and sanitation facilities), benefiting a population of more than 25 000 in the community mobilisation and incentivisation clusters, and the total cost contributed by the community was 36% against the target of 25% (appendix 3 p 10).

The endline survey was conducted on a total of 3812 children younger than 5 years after a 4% refusal rate: 1284 children from the community mobilisation and incentivisation group; 1276 children from the community mobilisation group, and 1252 children from the control group. At endline, 59·2% (380 of 657) children were fully immunised in the community mobilisation and incentivisation group, 43·7% (294 of 678) in the community mobilisation group, and 46·6% (294 of 662) in the control group (table 2). Use of oral rehydration solution was 53·3% (48 of 90) in the community mobilisation and incentivisation group, 37·9% (50 of 132) in the community mobilisation group, and 35·1% (47 of 134) in the control group, while mean sanitation index was 10·1 (SD 7·5) in the community mobilisation and incentivisation group, 9·3 (7·2) in the community mobilisation group, and 8·9 (7·4) in the control group. The results for the individual indicators of sanitation index are presented in appendix 3 (p 11). The multivariable analysis indicated that a higher proportion of children in the community mobilisation and incentivisation group were fully immunised than in the control group (adjusted RR 1·3 [95% CI 1·0–1·5]) but provided no evidence of a difference in proportion of fully immunised children when the community mobilisation group was compared with the control group (RR 0·9 [0·7–1·2]; table 3). The multivariable analysis suggests that the overall sanitation index (mean difference, 1·3 [95% CI 0·6–1·9]), drinking water index (mean difference, 0·4 [0·2–0·6]), and food index (mean difference, 0·7 [0·5–0·9]) were higher in the community mobilisation and incentivisation group than in the control group, but there was no evidence of a difference in sanitation index (mean difference, 0·4 [95% CI –0·1 to 1·0], drinking water index, food index or domestic household hygiene index between the community mobilisation group and the control group. The multivariable analysis suggests higher oral rehydration use in the community mobilisation and incentivisation group (adjusted RR 1·5 [95% CI 1·0–2·2]) than in the control group, while there was no evidence of a difference in oral rehydration use in the community mobilisation group (adjusted RR 0·9 [0·6–1·5]) compared with the control group.

**Table 3:** Multivariable analysis for primary and secondary outcomes compared with the control group

		**Unadjusted**				Adjusted*				**Intraclass correlation coefficient**
		Community mobilisation and incentivisation group	p value	Community mobilisation group	p value	Community mobilisation and incentivisation group	p value	Community mobilisation group	p value	
Sanitation Index†		1·2 (0·6 to 1·8)	<0·0001	0·4 (−0·2 to 0·9)	0·20	1·3 (0·6 to 1·9)	<0·0001	0·4 (−0·1 to 1·0)	0·16	0·375
	Drinking water index (score 0–3)	0·4 (0·2 to 0·6)	<0·0001	0·1 (−0·1 to 0·3)	0·36	0·4 (0·2 to 0·6)	<0·0001	0·1 (−0·1 to 0·3)	0·39	0·290
	Food index (score 0–3)	0·7 (0·4 to 0·9)	<0·0001	0·1 (−0·2 to 0·4)	0·39	0·7 (0·5 to 0·9)	<0·0001	0·2 (−0·1 to 0·5)	0·31	0·231
	Domestic household hygiene index (score 0–6)	0·1 (−0·2 to 0·4)	0·55	0·2 (−0·1 to 0·4)	0·21	0·1 (−0·2 to 0·4)	0·49	0·2 (−0·1 to 0·4)	0·17	0·436
Open defecation†		0·9 (0·7 to 1·3)	0·82	1·1 (0·9 to 1·4)	0·40	1·04 (0·8 to 1·3)	0·73	1·1 (0·9 to 1·4)	0·27	0·066
Children with diarrhoea during past 2 weeks‡		0·6 (0·5 to 0·9)	0·021	0·9 (0·7 to 1·3)	0·83	0·6 (0·5 to 0·9)	0·020	1·0 (0·7 to 1·4)	0·91	0·014
Care-seeking for childhood diarrhoea‡		0·9 (0·8 to 1·1)	0·31	0·8 (0·7 to 0·9)	0·027	0·9 (0·8 to 1·1)	0·33	0·8 (0·7 to 1·0)	0·068	0·035
Oral hydration solution use for childhood diarrhoea‡		1·5 (1·1 to 2·1)	0·012	1·1 (0·7 to 1·6)	0·71	1·5 (1·0 to 2·2)	0·036	0·9 (0·6 to 1·5)	0·77	0·066
Children with acute respiratory infection during past 2 weeks‡		0·8 (0·4 to 1·4)	0·43	0·8 (0·5 to 1·4)	0·52	0·8 (0·4 to 1·5)	0·45	0·8 (0·5 to 1·4)	0·43	0·019
Antibiotic treatment for childhood acute respiratory infection‡		0·8 (0·4 to 1·8)	0·73	0·7 (0·4 to 1·4)	0·48	0·9 (0·4 to 1·9)	0·21	0·8 (0·4 to 1·4)	0·99	0·110
Exclusive breastfeeding at 6 months‡		1·8 (1·4 to 2·3)	<0·0001	1·2 (0·9 to 1·7)	0·35	1·8 (1·4 to 2·3)	<0·0001	1·2 (0·9 to 1·7)	0·20	0·047
Age-appropriate vaccination‡										
	Fully immunised	1·3 (1·0 to 1·6)	0·031	0·9 (0·7 to 1·2)	0·63	1·3 (1·0 to 1·5)	0·030	0·9 (0·7 to 1·2)	0·64	0·099
	Partially immunised	0·8 (0·7 to 1·0)	0·10	1·1 (0·9 to 1·0)	0·50	0·8 (0·6 to 1·0)	0·076	1·1 (0·9 to 1·3)	0·55	0·036
	Not immunised	0·6 (0·3 to 1·1)	0·10	1·0 (0·6 to 1·7)	0·98	0·7 (0·4 to 1·2)	0·24	1·2 (0·9 to 1·5)	0·57	−0·077

RR=risk ratio.

* The estimates are adjusted for child sex, age, number of of siblings, mother's education, father's education, mother's occupation, father's occupation, lady health visitor visit, wealth quintiles, and household density.

† Indicates mean difference (95% CI).

‡ Indicates RR (95% CI).

For the secondary outcomes, the exclusive breastfeeding rate was 53·5% (333 of 622) in the community mobilisation and incentivisation group, 39·6% (256 of 647) in the community mobilisation group, and 36% (215 of 597) in the control group. The multivariable analysis suggested higher exclusive breastfeeding rates (RR 1·8 [95% CI 1·4–2·3]) in the community mobilisation and incentivisation group than in the control group, while there was no evidence for differences between the community mobilisation group and the control group. The prevalence of diarrhoea reduced in all clusters; 7·0% (90 of 1284) in the community mobilisation and incentivisation group; 10·3% (132 of 1276) in the community mobilisation group, and 10·7% (134 of 1252) in the control group. The prevalence of ARI reduced; 4·0% (51 of 1284) in the community mobilisation and incentivisation group, 4·2% (54 of 1276) in the community mobilisation group, and 5·0% (63 of 1252) in the control group. Use of antibiotics for acute respiratory infection treatment was highest in the community mobilisation and incentivisation group at 60·7% (31 of 51), 50·0% (27 of 54) in the community mobilisation group, and 57·1% (36 of 63) in the control group. Multivariable analysis suggests that diarrhoea prevalence in the community mobilisation and incentivisation group was lower than in the control group (adjusted RR 0·6 [95% CI 0·5–0·9]), while there was no evidence for differences between the community mobilisation and incentivisation group and the control group for acute respiratory infection prevalence. There was no evidence of difference in the prevalence of diarrhoea or acute respiratory infection in the community mobilisation group when compared with the control group.

The difference-in-differences analysis found evidence for an intervention effect in the community mobilisation and incentivisation group when compared with the control group for all primary and most secondary outcomes (appendix 3 p 13). There was no evidence for differences between the community mobilisation group and the control group except for exclusive breastfeeding, which was higher in the community mobilisation group.

## Discussion

The CoMIC trial evaluated a unique approach to community engagement and demand creation through community mobilisation and collective incentivisation to encourage uptake of essential child health interventions for children younger than 5 years. Findings from the CoMIC trial suggest that C3I, together with community mobilisation, leads to wider community acceptance, behaviour change, and significant improvement in all three primary outcomes including proportion of fully immunised children, oral rehydration solution use, and sanitation index. Exclusive breastfeeding rates and diarrhoea prevalence also improved in the community mobilisation and incentivisation group; while there was no evidence of an effect on any primary and secondary outcomes except the exclusive breastfeeding rate in the community mobilisation group when compared with the control group.

The coverage of essential child health interventions as well as care-seeking practices for childhood diarrhoea and pneumonia in Pakistan remain low.^10^ Previous studies assessing caregiver knowledge, perceptions, practices, and care-seeking behaviours related to childhood diarrhoea and pneumonia in Pakistan have highlighted factors including inaccessibility, lack of caregivers’ recognition of disease, home remedies, self-prescribed medicines, financial constraints, and low utilisation of community-based health services,^24^ and these findings align with the findings of our formative research.^19^ The CoMIC trial builds upon these findings and suggests that community mobilisation alone does not lead to behaviour change associated with improved uptake of essential interventions and care-seeking practices, unless tied to some tangible benefits. C3I could potentially act as an impetus for bringing change in community behaviours when these benefits are community driven, offered on serial incremental targets and conditioned on behaviour change, as demonstrated by all the clusters in the community mobilisation and incentivisation group meeting the targets for incentive eligibility at the second and third evaluation. Results from the CoMIC trial suggest that the community is ready to invest if they decided on the incentives, as was evident in the 36% of cost sharing against the target of 25% through providing labour, land, and supplies. These community-driven incentives delivered in the CoMIC trial included mostly water and sanitation facilities (hand pumps, water supply lines, and latrines), which not only promoted ownership and sustainability but can have multifold benefits by improving the general sanitation and health status and wellbeing of the communities.

Community mobilisation and incentives to improve child health outcomes have been evaluated previously.^13^, ^14^ Individual conditional and unconditional incentives, user-directed as well as provider-directed, have shown modest and variable improvements in coverage of reproductive, maternal, and child health indicators, but there is scepticism regarding their sustainability and relative cost-effectiveness.^14^ Moreover, there is lack of evidence on the relative effectiveness and efficiency of various incentive types and their long-term sustainability.^14^ Although there is considerable momentum in support of incorporating community mobilisation activities in maternal and child health programmes, there is limited evidence on the processes, operationalisation, and evaluation and only a few studies seek to elucidate the mechanism.^13^ Community mobilisation can affect different populations in different ways with no straightforward mechanisms, and there is a lot of advocacy for collaborations between public health and behaviour change experts, specifically regarding WASH behaviours^13^, ^15^, ^25^ and to help communities understand the impact on broader community wellbeing.^17^ The findings of three large trials of unprecedented scale and cost found no effect of basic WASH interventions on childhood stunting, and only mixed effects on childhood diarrhoea and support a call for transformative WASH—a comprehensive package of WASH interventions that is tailored to address the local exposure landscape and enteric disease burden.^26^ A relatively recent approach, community-led total sanitation, focuses on behaviour change related to open defecation and a systematic review on the effectiveness of this approach suggests reduction in open defecation; however, there is a need for further rigorous research on the quantitative effects of community-led total sanitation as well as addressing implementation challenges.^27^

The CoMIC trial provides important insights for designing context-specific community-directed interventions for child health and our formative work was in line with the recent developments. The findings reinforce that although behaviours are often difficult to change, change is achievable if the community is provided with tangible short-term incentives and has the autonomy to lead the mobilisation activities and choose the incentive according to their needs and priorities.^20^ The collective nature of the mobilisation in a closely interlinked society makes persuasion of resistant households easier and helps them to discuss and deliberate and make health and hygiene a priority. The CoMIC trial utilised a composite index to measure household sanitation status through spot checks, as there is growing evidence around the composite indices being more stable over time compared with individual indicators and the sanitation index used in this trial has been reported to be associated with a reduction in diarrhoea.^22^

There are a few limitations of this study; first, the strategy of community mobilisation and incentivisation was tested for a defined number of outcomes in a defined population and would need further studies on various outcomes and in different contexts. Second, this required serial monitoring and evaluation which might require further economic evaluations and cost considerations before wider implementation. However, such serial monitoring mechanisms could potentially strengthen accountability which is urgently needed in low-income countries. Third, for sanitation index, we did not compare practices assessed by spot checks to practices assessed by other measures, because indicators of hygiene practices assessed by spot checks are subject to substantial day-to-day variations. Although composite indices using multiple indicators to capture aspects of hygienic behaviour might not allow the assessment of the effect of a specific practice, using multiple indicators can capture several practices at the same time, that might influence the overall risk for diarrhoea. Moreover, factors influencing childhood diarrhoea in children from low-resource settings are multilayered and hence no conclusion can be made regarding a possible reduction in pathogen exposure. Finally, we acknowledge that one of our primary outcomes (oral rehydration solution use) was self-reported and hence there was a chance of over-reporting due to incentivisation. Nonetheless, two of our primary outcomes (proportion of fully immunised children and sanitation index) were not self-reported and based on data collected by independent field teams to ensure accuracy and reliability. Immunisation status was ascertained via vaccination cards, for cases in which this was available as for more than 75% of the children in the endline survey. Sanitation index for each household was independently assessed by two fieldworkers from each household to ensure a more objective measurement. Furthermore, it is noteworthy that exclusive breastfeeding rates were not one of the incentivised indicators; however, improvements of this secondary outcome in both the community mobilisation and incentivisation and community mobilisation groups were observed, compared with control. Efforts were made to ensure that the clusters in different study groups were not geographically close; however, some households in the control clusters could have had access to the educational materials distributed in the intervention clusters.

The CoMIC trial provides rigorous and pragmatic evidence as it was conducted on a large scale in a population of around 150 000 people and spanned over 2 years. The trial was conducted in a strategic geographical location of Pakistan, a country with one of the highest childhood morbidity and mortality rates attributable to diarrhoea and pneumonia globally. However, there is a need to explore sustainability of the effective intervention in the CoMIC trial like any other public health innovation.^28^ The CoMIC trial had some built-in features to ensure sustainability in the design phase: for example, incorporating formative research to understand community motives and emotions that led to community itself being a driver and supporter of behaviour change, and the community further contributing towards labour and land provision for the incentives that translated into community ownership of the incentive and potential future sustainability. However, further studies will be needed to assess the cost-effectiveness and long-term sustainability of such strategies, and these evaluations are in process and will be published as separate papers. The community mobilisation and collective incentivisation strategy could be scaled up and in the context of Pakistan, it can utilise the lowest administrative unit which is the Union Council. The Union Councils already have an elected committee and an annual budget of their own and most of these funds are spent on providing WASH facilities. The community engagement and demand creation strategy evaluated in the CoMIC trial could be replicated at the Union Council or the lowest administrative level to improve health-seeking behaviours at the community level and consequently improving utilisation of these WASH facilities. Pakistan also has an unconditional cash transfer programme (Benazir Income Support Program) and this strategy could be tied to such programmes to yield synergistic effects on health and development and increase accountability at the lowest administrative unit.

The community mobilisation and incentivisation strategy utilised in the CoMIC trial led to enhanced acceptance of evidence-based child health interventions, as evidenced by the increased uptake of essential interventions for child health including immunisation, use of oral rehydration solution, and exclusive breastfeeding. The findings from the CoMIC trial have the potential to inform policy and future implementation of programmes targeting behaviour change and provision of incentives, but would need to be evaluated for varying outcomes in different contexts.

## Contributors

## Equitable partnership declaration

## Data sharing

The study protocol is published and referenced in the manuscript. Individual participant data that underlie the results reported in this Article can be shared after de-identification (text, tables, figures, and appendices) immediately after publication up to 5 years following publication. Data would be made available to researchers who provide a methodologically sound proposal. Proposals should be directed to the corresponding author and to gain access, data requestors will need to sign a data access agreement.

## Declaration of interests

We declare no competing interests.

## Supplementary Materials

Sindhi translation of the abstract

Urdu translation of the abstract

Supplementary appendix 3

Supplementary appendix 4 Equitable Partnership Declaration
