# Supplementary material for: An innovative Community Mobilisation and Community Incentivisation for child health in rural Pakistan (CoMIC): a cluster-randomised, controlled trial
Source: Lancet Glob Health. 2024 Dec 18;13(1):e121–33. doi: 10.1016/S2214-109X(24)00428-5 (PMC11659842; doi:10.1016/S2214-109X(24)00428-5)
Supplement: Sindhi translation of the abstract [file mmc1.pdf]

THE LANCET
Global Health

Supplementary appendix 1

This translation in Sindhi was submitted by the authors and we reproduce it as supplied. It has not been peer reviewed. *The Lancet*'s editorial processes have only been applied to the original in English, which should serve as reference for this manuscript.

هي سنڌيءَ ترجمو ليکڪن پاران جمع ڪرايو ويو آهي ۽ اسان ان ترجمي کي جيئن جمع ڪرايو ويو آهي تيئن ئي ٻيهر پيش ڪيو آهي- ان ترجمي جي ساٿي ماهرن پاران ڪنهن به قسم جي نظرثاني نه ڪئي وئي آهي- لانسيٽ جو ايڊيٽوريل عمل / ڪارروائيون صرف انگريزيءَ مسودي تي ڪئي وئي آهي جيڪي هن مسودي لاءِ حوالو طور ڪم ڪرڻ گهرجي-

Supplement to: Das JK, Salam RA, Padhani ZA, et al. An innovative Community Mobilisation and Community Incentivisation for child health in rural Pakistan (CoMIC): a cluster-randomised, controlled trial. *Lancet Glob Health* 2025; **13:** e121–33.

پاڪستان ۾ ٻارن ۾ دستن ۽ نمونيا جي روڪٿام لاءِ ثبوتن جي بنياد تي ڪميونٽي کي متحرڪ ڪرڻ ۽ تَرغِيبون ڏيڻ واري حڪمت عملي جو جائزو

پس منظر:

ٻارن جي صحت جي بهتري جي بنيادي سرگرمين /قدمن تائين رسائي ۽ قبوليت ۾ فرق جي ڪري وچڙندڙ بيماريون اڃان تائين پنج سالن کان گهٽ / ننڍي عمر جي ٻارن جي موت جو وڏو اهم سبب آهن. ثبوتن جي بنياد تي پاڪستان ۾ ٻارن جي صحت جي بهتري جي بنيادي سرگرمين/ قدمن جي ڪوريج کي بهتر ڪرڻ لاءِ ڪميونٽي کي متحرڪ ڪرڻ ۽ تَرغِيبون ڏيڻ واري حڪمت عملي جو جائزو ڪيو ويو هو.

طريقہ ڪار:

هي هڪ اِمڪاني طريقي سان چونڊيل ۽ ضابطي هيٺ ڪلسٽر قسم جو مطالعو (ڪلسٽر رينڊمائيزڊ ڪنٽرول ٽرائل) هو، جيڪو آڪٽوبر 2018 کان آڪٽوبر 2020 جي دوران پاڪستان جي ضلعي ٽنڊي محمد خان جي ٻهراڙي / ديهي / ڳوٺن ۾ ڪيو ويو هو. هن مطالعي ۾ ٻن قسمن جون سرگرميون شامل هيون: هڪ ڪميونٽي کي متحرڪ ڪرڻ ۽ ٻيون ڪميونٽي کي تَرغِيبون ڏيڻ واريون. برادري کي متحرڪ ڪرڻ لاءِ ڳوٺاڻيون ڪميٽيون تشڪيل ڏنيون ويون، جن ڳوٺاڻن لاءِ دستن ۽ نمونيا جي روڪٿام جي باري ۾ آگاهي واريون سرگرميون منعقد ڪيون، جڏهن تہ تَرغِيبون شرط جي بنياد، ڳَڏيل/اجتماعي، ۽ ڪميونٽي سطح تي ڏنيون ويون هيون. هن مطالعي ۾ جاگرافيائي ويجهڙائي/نزديڪي ۽ نسلي هڪجهڙائي جي بنياد تي ڳوٺن کي ملائي /گڏي ڪل 48 ڪلسٽر ٺاهيا ويا ۽ هر ڪلسٽر جي آبادي 1500 کان 3000 رکي وئي. هن مطالعي ۾ ڪل 24,846 گهراڻن جي ڪل آبادي 139,005 شامل ڪئي وئي جن ۾ پنج سالن کان ننڍي/ گهٽ عمر وارا 21,638 ٻار بہ شامل هئا. ڪلسٽرن کي 1:1:1 جي نسبت سان اِمڪاني طريقي سان ٽن گروپن ۾ ورهايو ويو:

1. پهرين گروپ جي ڪلسٽرن ۾ ٻئي سرگرميون ڪميونٽي کي متحرڪ ڪرڻ ۽ ڪميونٽي کي تَرغِيبون ڏيڻ واريون ڪيون ويون.
2. ٻي گروپ جي ڪلسٽرن ۾ صرف ڪميونٽي کي متحرڪ ڪرڻ واريون سرگرميون ڪيون ويون.
3. ۽ جڏهن تہ ٽئين گروپ (ضابطي هيٺ/ڪنٽرول گروپ) جي ڪلسٽرن ۾ ڪابہ سرگرمي نہ ڪئي وئي.

پهرين گروپ ۾ 152 ڳوٺن جا پنج سالن کان ننڍي/ گهٽ عمر وارا 7361 ٻار شامل هئا، ٻيو گروپ 166 ڳوٺن ۽ 7546 پنج سالن کان ننڍي/ گهٽ عمر واري ٻارن تي مشتمل هو، ۽ جڏهن تہ ٽئين گروپ ۾ 139 ڳوٺن جا پنج سالن کان ننڍي/ گهٽ عمر وارا 6731 ٻار شامل هئا. ڪلسٽرن کي تَرغِيبون ڏيڻ لاءِ مطالعي جي بنيادي/ مکيہ نتيجن ۾ مسلسل/ ترتيبوار ڇهن مهينن، پندرهن، ۽ چويهن مهينن تي ڪلسٽر جي سطح تي بهتري ٿيڻ هڪ شرط رکي ويئي هئي. ڪلسٽرن کي غير نقدي تَرغِيبون ڏيڻ جو فيصلو ڳوٺاڻين ڪميٽين پاران ڪيو ويو هو. هن مطالعي جي بنيادي/ مکيہ نتيجن ۾ سيئي حفاظتي ٽڪا لڳرايل ٻارن جو تناسب/تعداد، او آر ايس (ORS) جي استعمال جي تناسب/تعداد، ۽ صفائيءَ جي مجموعي معيار جي تناسب/تعداد شامل ڪيو ويو هو. هن مطالعي جي اَنگَ اکرَ /اعداد شمار (ڊيٽا) جو تفصيلي تَجزيو هڪ آزاد ٽيم پاران ڪيو ويو جيڪا هن مطالعي جي گروپن کان بي خبر هئي. هن مطالعي جي آخر واري تجزئي ۾ 3812 ٻار شامل ڪيا ويا (1284 ڪميونٽي کي متحرڪ ڪرڻ ۽ تَرغِيبون ڏيڻ واري گروپ جا، 1276 ڪميونٽي کي متحرڪ ڪرڻ واري گروپ جا، ۽ 1252 ڪنٽرول گروپ جا). هي مطالعو ClinicalTrials.gov تي رجسٽرڊ ٿيل آهي جنهن جو نمبر NCT03594279 آهي.

نتيجا:

گهڻن متغيرن واري تجزئي (ملٽي ويريبل اينالائسس) مان ظاهر ٿيو تہ ڪميونٽي کي متحرڪ ڪرڻ ۽ تَرغِيبون ڏيڻ واري گروپ جي ڪلسٽرن ۾ مطالعي جي سيني بنيادي/ مکيہ نتيجن ۾ واضح بهتري آئي آهي. سرگرمين جي چويهن مهينن کان پوءِ ڪنٽرول گروپ جي ڪلسٽرن جي ڀيٽ/مقابلي ۾ ڪميونٽي کي متحرڪ ڪرڻ ۽ تَرغِيبون ڏيڻ واري گروپ جي ڪلسٽرن ۾ سيئي حفاظتي ٽڪا لڳرايل ٻارن جو تناسب/تعداد وڌيڪ هئو (risk ratio [RR] 1·3 [95% CI 1·0—1·5])، او آر ايس (ORS) جي استعمال جي تناسب/تعداد ۾ اضافو (RR 1·5 [1·0—2·2])، ۽ صفائيءَ جي مجموعي معيار جي تناسب/تعداد ۾ واڌارو (RR 1·5 [1·0—2·2]) نمايان طور ڏٺو ويو. جڏهن تہ ڪميونٽي کي متحرڪ ڪرڻ واري گروپ جي ڪلسٽرن ۽ ڪنٽرول گروپ جي ڪلسٽرن جي وچ ۾ اهڙو واضح فرق ڏسڻ ۾ نہ آيو.

تشريح/ سمجهاڻي:

ڪميونٽي کي متحرڪ ڪرڻ ۽ تَرغِيبون ڏيڻ واري سرگرمين جي ڪري ٻارن جي صحت لاءِ ضروري قدمن جي ڪميونٽي ۾ قبوليت بهتر ٿي، جيڪا ڪميونٽي جي روئين ۾ بهتري ۽ ٻارن جي صحت لاءِ ضروري قدمن جي ڪوريج ۾ بهتري سان ظاهر ٿيئي ٿي. هن مطالعي جا نتيجا ڪميونٽي جي روين ۾ تبديلي آڻڻ لاءِ پاليسي ناهڻ ۽ مستقبل ۾ اهڙن پروگرامن جي عملدرآمد لاءِ رهنمائي فراهم ڪرڻ جي صلاحيت رکن ٿا.
