# Supplementary material for: An innovative Community Mobilisation and Community Incentivisation for child health in rural Pakistan (CoMIC): a cluster-randomised, controlled trial
Source: Lancet Glob Health. 2024 Dec 18;13(1):e121–33. doi: 10.1016/S2214-109X(24)00428-5 (PMC11659842; doi:10.1016/S2214-109X(24)00428-5)
Supplement: Urdu translation of the abstract [file mmc2.pdf]

THE LANCET
Global Health

## Supplementary appendix 2

This translation in Urdu was submitted by the authors and we reproduce it as supplied. It has not been peer reviewed. *The Lancet*'s editorial processes have only been applied to the original in English, which should serve as reference for this manuscript.

اردو میں یہ ترجمہ مصنفین کے ذریعہ پیش کیا گیا تھا اور ہم اس کی ہو بہو دوبارہ تخلیق کرتے ہیں۔ اس کا ہم مرتبہ ماہروں کے ذریعہ جائزہ نہیں لیا گیا ہے۔ *لانسیٹ* کے ادارتی عمل کا اطلاق صرف انگریزی کے اصل پر ہوا ہے، جو اس نسخے کے حوالہ کے طور پر کام کرے گا

Supplement to: Das JK, Salam RA, Padhani ZA, et al. An innovative Community Mobilisation and Community Incentivisation for child health in rural Pakistan (CoMIC): a cluster-randomised, controlled trial. *Lancet Glob Health* 2025; **13:** e121–33.

# پاکستان میں بچوں میں اسہال اور نمونیا کی روک تھام کے لیے شواہد پر مبنی کمیونٹی کو متحرک کرنے اور ترغیبات / مراعات فراہم کرنے کی حکمت عملیوں کا جائزہ


## پس منظر:

بچوں کی صحت کو بہتر بنانے کے لیے بنیادی اقدامات تک رسائی اور قبولیت میں فرق کے باعث وبائی بیماریاں اب بھی پانچ سال سے کم عمر کے بچوں کی اموات کی ایک اہم وجہ ہیں۔ پاکستان میں بچوں کی صحت کو بہتر بنانے کے لیے ثبوتوں پر مبنی بنیادی اقدامات کی کوریج کو بہتر بنانے کے لیے کمیونٹی کو متحرک کرنے اور ترغیبات فراہم کرنے کی حکمت عملی کا جائزہ لیا گیا تھا۔

## طریقہ کار:

یہ ایک امکانی انتخاب کردہ اور ضابطے کے تحت کلسٹر پر مبنی مطالعہ (کلسٹر رینڈمائزڈ کنٹرول ٹرائل) تھا، جو اکتوبر 2018 سے اکتوبر 2020 کے دوران پاکستان کے ضلع ٹنڈو محمد خان کے دیہی علاقوں میں کیا گیا تھا۔ اس مطالعے میں دو قسم کی سرگرمیاں شامل تھیں: ایک کمیونٹی کو متحرک کرنا اور دوسری کمیونٹی کو ترغیبات / مراعات فراہم کرنا۔ کمیونٹی کو متحرک کرنے کے لیے گاؤں کی کمیٹیاں تشکیل دی گئیں، جنہوں نے گاؤں کے بچوں کے لیے اسہال اور نمونیا کی روک تھام کے بارے میں آگاہی کی سرگرمیاں منعقد کیں، جبکہ ترغیبات / مراعات شرط کی بنیاد پر، اجتماعی، اور کمیونٹی سطح پر فراہم کی گئیں۔ اس مطالعے میں گاؤں کو جغرافیائی قربت اور نسلی مطابقت کی بنیاد پر ملا کر مجموعی طور پر 48 کلسٹر بنائے گئے، اور ہر کلسٹر کی آبادی 1500 سے 3000 کے درمیان رکھی گئی۔ اس مطالعے میں مجموعی طور پر 24,846 گھروں کی کل آبادی 139,005 شامل کی گئی، جن میں پانچ سال سے کم عمر کے 21,638 بچے بھی شامل تھے۔ کلسٹرز کو 1:1:1 کے تناسب سے امکانی (رینڈم) طریقے سے تین گروپوں میں تقسیم کیا گیا:

1. پہلے گروپ کے کلسٹرز میں دونوں سرگرمیاں، یعنی کمیونٹی کو متحرک کرنے اور کمیونٹی کو ترغیبات / مراعات فراہم کرنے کی گئیں۔
2. دوسرے گروپ کے کلسٹرز میں صرف کمیونٹی کو متحرک کرنے کی سرگرمیاں کی گئیں۔
3. جبکہ تیسرے گروپ (کنٹرول گروپ) کے کلسٹرز میں کوئی سرگرمی نہیں کی گئی۔

پہلے گروپ میں 152 گاؤں کے پانچ سال سے کم عمر کے 7361 بچے شامل تھے، دوسرا گروپ 166 گاؤں اور 7546 پانچ سال سے کم عمر کے بچوں پر مشتمل تھا، جبکہ تیسرے گروپ میں 139 گاؤں کے پانچ سال سے کم عمر کے 6731 بچے شامل تھے۔ کلسٹرز کو ترغیبات / مراعات فراہم کرنے کے لیے مطالعے کے بنیادی نتائج میں سلسلہ وار چھ ماہ، پندرہ ماہ، اور چوبیس ماہ پر کلسٹر کی سطح پر بہتری کی شرط رکھی گئی تھی۔ کلسٹرز کو غیر نقدی ترغیبات / مراعات فراہم کرنے کا فیصلہ گاؤں کی کمیٹیوں کی جانب سے کیا گیا تھا۔ اس مطالعے کے بنیادی نتائج میں مکمل حفاظتی ٹیکے لگوانے والے بچوں کا تناسب، او آر ایس (ORS) کے استعمال کا تناسب، اور صفائی کے مجموعی معیار کا تناسب شامل کیا گیا تھا۔ اس مطالعے کے اعداد و شمار (ڈیٹا) کا تفصیلی تجزیہ ایک آزاد ٹیم نے کیا، جو اس مطالعے کے گروپوں سے لاعلم (بلائنڈڈ) تھی۔ مطالعے کے آخری تجزیے میں 3812 بچے شامل کیے گئے (1284 کمیونٹی کی متحرک اور ترغیبات / مراعات فراہم کرنے والے گروپ کے، 1276 کمیونٹی کو متحرک کرنے والے گروپ کے، اور 1252 کنٹرول گروپ کے)۔ یہ مطالعہ ClinicalTrials.gov پر رجسٹرڈ ہے، جس کا نمبر NCT03594279 ہے۔

## نتائج:

کثیر متغیر کے تجزیے (ملٹی ویریٹ اینالائسس) سے معلوم ہوا کہ کمیونٹی کو متحرک کرنے اور ترغیبات فراہم کرنے والے گروپ کے کلسٹرز میں مطالعے کے تمام بنیادی نتائج میں واضح بہتری دیکھنے میں آئی ہے۔ سرگرمیوں کے 24 ماہ کے بعد، کنٹرول گروپ کے کلسٹرز کے مقابلے میں، کمیونٹی کو متحرک کرنے اور ترغیبات فراہم کرنے والے گروپ کے کلسٹرز میں مکمل حفاظتی ٹیکے لگوانے والے بچوں کا تناسب زیادہ تھا (risk ratio [RR] 1·3 [95% CI 1·0–1·5])، او آر ایس (ORS) کے استعمال کے تناسب میں اضافہ (RR 1·5 [1·0–2·2])، اور صفائی کے مجموعی معیار میں بہتری (RR 1·5 [1·0–2·2]) نمایاں طور پر دیکھی گئی۔ تاہم، صرف کمیونٹی کو متحرک کرنے والے گروپ کے کلسٹرز اور کنٹرول گروپ کے کلسٹرز کے درمیان ایسا کوئی واضح فرق دیکھنے میں نہیں آیا۔

## تشریح:

کمیونٹی کو متحرک کرنے اور ترغیبات / مراعات فراہم کرنے والی سرگرمیوں کی وجہ سے بچوں کی صحت کے لیے ضروری اقدامات کی کمیونٹی میں قبولیت بہتر ہوئی، جو کمیونٹی کے رویوں میں بہتری اور بچوں کی صحت کے لیے ضروری اقدامات کی کوریج میں اضافے سے ظاہر ہوتی ہے۔ اس مطالعے کے نتائج کمیونٹی کے رویوں میں تبدیلی لانے کے لیے پالیسی سازی اور مستقبل میں ایسے پروگراموں کے نفاذ کے لیے رہنمائی فراہم کرنے کی صلاحیت رکھتے ہیں۔
