# Supplementary material for: An innovative Community Mobilisation and Community Incentivisation for child health in rural Pakistan (CoMIC): a cluster-randomised, controlled trial
Source: Lancet Glob Health. 2024 Dec 18;13(1):e121–33. doi: 10.1016/S2214-109X(24)00428-5 (PMC11659842; doi:10.1016/S2214-109X(24)00428-5)
Supplement: Supplementary appendix 3 [file mmc3.pdf]

THE LANCET
Global Health

Supplementary appendix 3

This appendix formed part of the original submission and has been peer reviewed. We post it as supplied by the authors.

Supplement to: Das JK, Salam RA, Padhani ZA, et al. An innovative Community Mobilisation and Community Incentivisation for child health in rural Pakistan (CoMIC): a cluster-randomised, controlled trial. *Lancet Glob Health* 2025; **13:** e121–33.

Supplementary Appendix

Figure 1: Theoretical Model of the CoMIC trial based on the Theory of Reasoned Action and Theory of Planned Behaviour

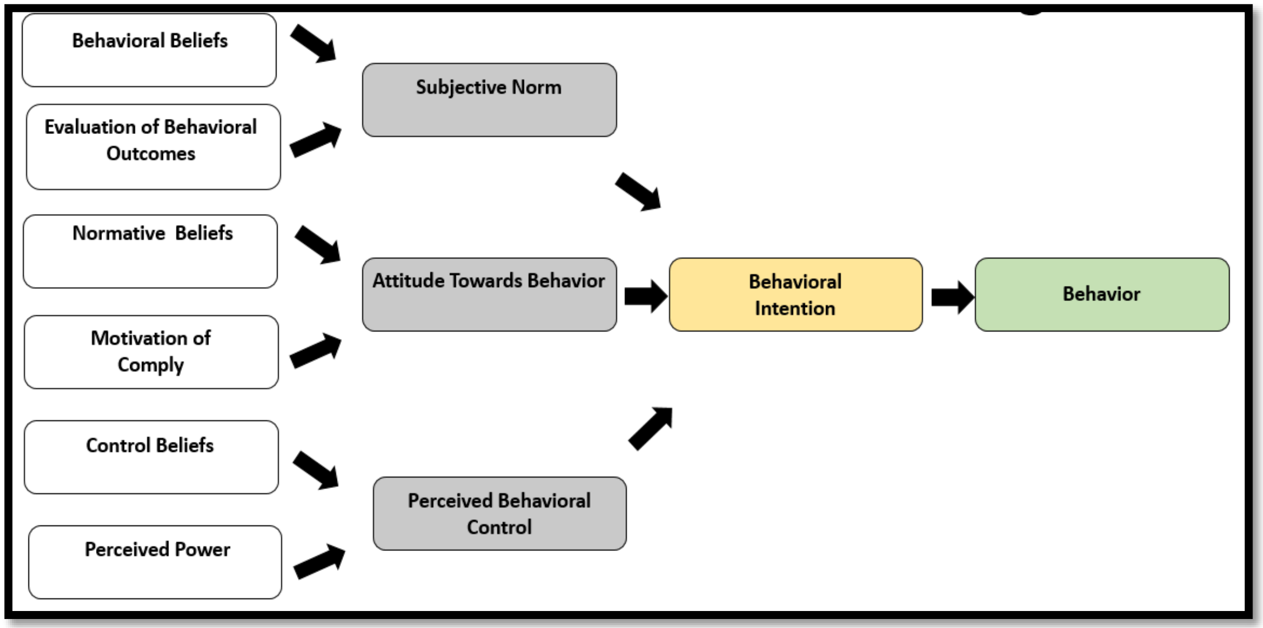

Figure 2a: Geographic distribution of population in the district

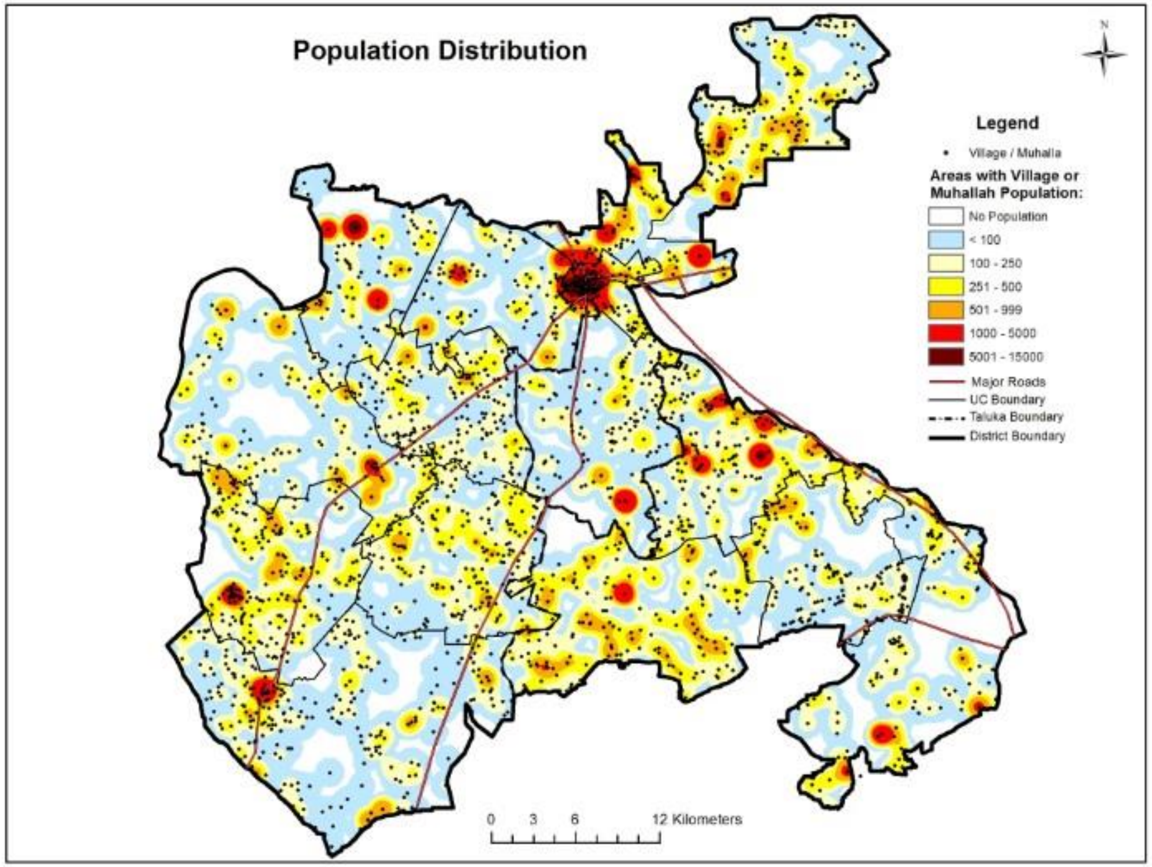

Figure 2b: Geographic distribution of clusters by study arms

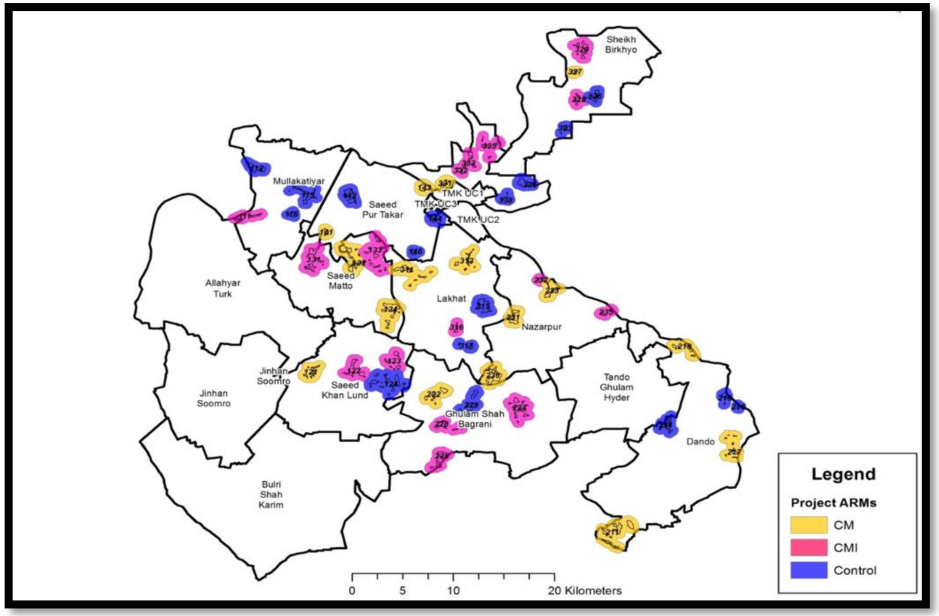

*CM=Community Mobilization; CMI=Community Mobilization and Incentivization

Figure 3: Activities and Timelines

Activity	Pre-Intervention Roll Out		Intervention Roll-Out Year 1		Intervention Roll-Out Year 2	
	Jan 2018 – Jun 2018	Jul 2018 – Sep 2018	Oct 2018 – Mar 2019	Apr 2019 - Dec 2020	Jan 2020 – Oct 2020	Nov 2020 – Dec 2020
Formative Research - Geographic information system mapping of the study area; baseline household survey; focus group discussions, and in-depth interviews						
Randomisation						
Identification and formation of village committees in CMI and CM arms						
Training and education to the committees						
Roll out of intervention						
Serial surveys and incentive provision in CMI arm						
End line Survey						
Final incentive delivery						

*CMI: Community Mobilization and Incentivization; CM: Community Mobilization

Figure 4: Summary of Need Assessment, Prioritization, and Incentive Delivery

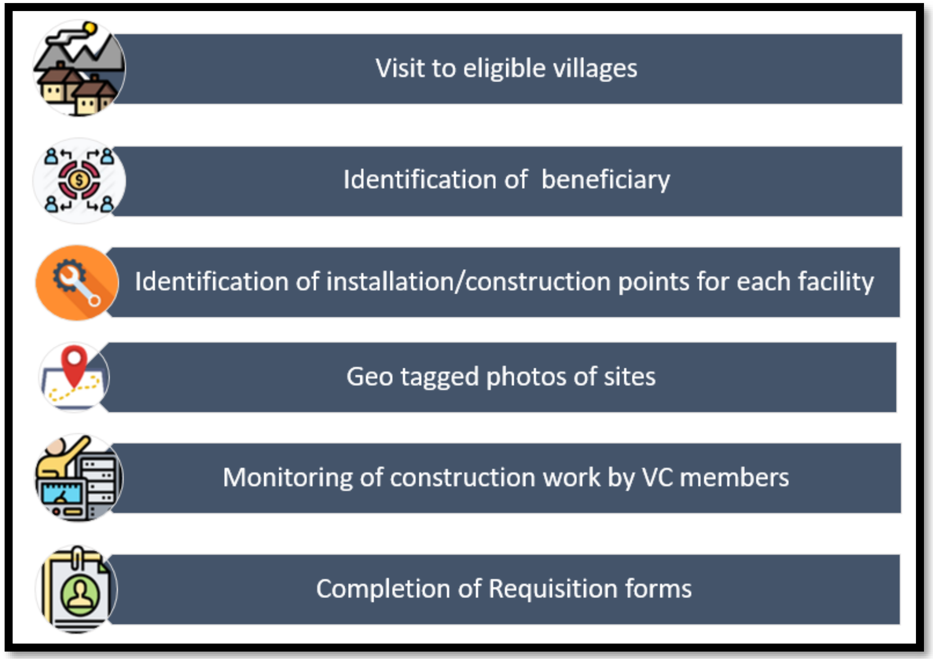

Table 1: Community incentives identified in need prioritizing meetings

Facility	Feasibility/Justification
Simple hand pumps	Where ground water was sweet - simple hand pumps were provided.
Lead line # of hand pumps	Where the ground water within the village/muhalla was brackish, a hand pump with horizontal lead line was installed from a sweet water source (maximum 1000 feet).
Lead line # of Solar motor pumps	Where the ground water within the village/muhalla was brackish, and distance of sweet water source was more than 1000 feet, a solar motor pump was installed with lead line to provide drinking water in village
Complete Toilet	The village in which people were more marginalized and couldn't afford the cost of super structure, project provided them complete toilets.
Toilet Sub Structure	The village in which people could afford and were willing to construct super structures on their own, project provided them only sub-structure of toilets with pit, this also increased the number of facilities. At the cost of one complete toilet - two sub-structures could be built.
Earth Filling & Main gate of school	Only one cluster with two settlements decided to use their incentive award on its school. They identified the filling of the school grounds and the construction of a main gate at the school as priority needs."

Table 2: Indices and items in the sanitation index

Indices and items in the sanitation index	
Indices	Items
Drinking water index<br>Possible score : 0-3	Interior water container water is covered<br>Exterior water container is clean<br>Container contains water
Food index<br>Possible score : 0-3	Clean dishes are covered<br>Clean dishes are stored high<br>All food is covered
Domestic hygiene<br>Possible score : 0-6	Absence of trash (inside/outside house)<br>No unrestrained animal in house<br>No accumulation of dirty clothes<br>Insignificant number of flies in house<br>No standing water in around house

Table 3: Sections of the Data Collection Tool

Sections included in the data collection tool
Section A: Geo-Spatial location coordinates
Section B: Household identification and demographic information
Section C: Introduction and consent
Section D: Household members' information
Section E: Socio economic status of household
Section F: Reproductive health, maternal and child mortality
Section G: Child health (diarrhea)
Section H: Child health (acute respiratory infection (ARI))
Section I: Immunization
Section J: Breast feeding and nutrition
Section K: Water and sanitation
Section L: Handwashing
Section M: Sanitation index (spot check)

Table 4: Incentives give at each assessment

Incentive Phase	Clusters	Simple of HPs	L. Line HPs	L. Line E. Motors	Water Storage Tanks	Complete Toilet	Complete Toilet with HW Tank	Super-Structure Toilet	School Work	Total Facilities
**Phase-1**	11	29	13	03	-	103	-	25	01	174
**Phase-2**	16	61	4	-	07	163	03	7	03	248
**Phase-3**	16	-	-	-	-	96	-	-	-	96
**Total**		90	17	03	07	362	03	32	04	518

Table 5: Total Incentives, Beneficiaries and Cost

Incentive/ Facility	No of Facilities	Beneficiary HHs	Beneficiary Individuals	Project contribution (PKR)	Community Contribution (PKR)	Total Cost	Project contribution in %.	Community Contribution in %.
Drinking Water	***117***	***1,970***	***12,788***	***1,333,049***	***1,204,800***	***2,537,849***	***53%***	***47%***
Simple Hand Pump	90	1,067	7,230	532,338	894,415	1,426,753	37%	63%
Hand Pump with Lead-line	17	430	2,566	460,521	153,635	614,156	75%	25%
Electric Motor with lead line	3	233	1,528	239,366	83,250	322,616	74%	26%
Water Storage Tanks	7	240	1,464	100,824	73,500	174,324	58%	42%
Sanitation	***397***	***1,914***	***12,392***	***9,256,254***	***4,895,295***	***14,151,549***	***65%***	***35%***
Complete Toilet	362	1,744	11,304	8,818,151	4,361,750	13,179,901	67%	33%
Complete toilet with HW Tank	3	15	99	81,570	38,000	119,570	68%	32%
Toilet Sub-Structure	25	124	794	253,680	425,705	679,385	37%	63%
Toilet Super-Structure	7	31	195	102,853	69,840	172,693	60%	40%
School Work	***4***	***3***	***71***	***292,083***	***25,000***	***317,083***	***92%***	***8%***
**Grand Total**	**518**	**3,887**	**25,251**	**10,881,387**	**6,125,095**	**17,006,482**	**63.98%**	**36.02%**

Table 6: Results of the individual indicator of sanitation index (n(%))

Sanitation Index Indicators	Baseline				Endline			
	Total	CMI	CM	C	Total	CMI	CM	C
	N=5,348	N=1,694	N=1,817	N=1,837	N=3,812	N=1,284	N=1,276	N=1,252
Water storage container covered?								
No	1,115 (20.8)	356 (21.0)	405 (22.3)	354 (19.3)	756 (20.9)	256 (20.0)	345 (27.6)	155 (14.2)
Yes	4,233 (79.2)	1,338 (79.0)	1,412 (77.7)	1,483 (80.7)	2,862 (79.1)	1,022 (80.0)	906 (72.4)	934 (85.8)
Water storage container cleaned?								
No	2,695 (50.4)	889 (52.5)	949 (52.2)	857 (46.7)	1,656 (45.8)	359 (28.1)	607 (48.5)	690 (63.4)
Yes	2,653 (49.6)	805 (47.5)	868 (47.8)	980 (53.3)	1,962 (54.2)	919 (71.9)	644 (51.5)	399 (36.6)
Water present in water storage container?								
No	1,114 (20.8)	362 (21.4)	368 (20.3)	384 (20.9)	135 (3.7)	11 (0.9)	86 (6.9)	38 (3.5)
Yes	4,234 (79.2)	1,332 (78.6)	1,449 (79.7)	1,453 (79.1)	3,483 (96.3)	1,267 (99.1)	1,165 (93.1)	1,051 (96.5)
Dishes/utensils are kept high?								
No	4,397 (82.2)	1,426 (84.2)	1,482 (81.6)	1,489 (81.1)	778 (21.5)	77 (6.0)	334 (26.7)	367 (33.7)
Yes	951 (17.8)	268 (15.8)	335 (18.4)	348 (18.9)	2,840 (78.5)	1,201 (94.0)	917 (73.3)	722 (66.3)
Dishes/utensils are kept high?								
No	3,144 (58.8)	1,021 (60.3)	1,085 (59.7)	1,038 (56.5)	814 (22.5)	78 (6.1)	354 (28.3)	382 (35.1)
Yes	2,204 (41.2)	673 (39.7)	732 (40.3)	799 (43.5)	2,804 (77.5)	1,200 (93.9)	897 (71.7)	707 (64.9)
All food is covered?								
No	1,990 (37.2)	679 (40.1)	654 (36.0)	657 (35.8)	2,756 (76.2)	905 (70.8)	995 (79.5)	856 (78.6)
Yes	3,358 (62.8)	1,015 (59.9)	1,163 (64.0)	1,180 (64.2)	862 (23.8)	373 (29.2)	256 (20.5)	233 (21.4)
No trash outside house								
No	1,241 (23.2)	438 (25.9)	423 (23.3)	380 (20.7)	2,010 (55.6)	1,005 (78.6)	558 (44.6)	447 (41.0)
Yes	4,107 (76.8)	1,256 (74.1)	1,394 (76.7)	1,457 (79.3)	1,608 (44.4)	273 (21.4)	693 (55.4)	642 (59.0)
No trash inside house								
No	2,570 (48.1)	828 (48.9)	838 (46.1)	904 (49.2)	2,917 (80.6)	1,224 (95.8)	928 (74.2)	765 (70.2)
Yes	2,778 (51.9)	866 (51.1)	979 (53.9)	933 (50.8)	701 (19.4)	54 (4.2)	323 (25.8)	324 (29.8)
Is there unrestrained animal in patio/house?								
Yes	1,658 (31.0)	560 (33.1)	482 (26.5)	616 (33.5)	199 (5.5)	20 (1.6)	66 (5.3)	113 (10.4)
No	3,690 (69.0)	1,134 (66.9)	1,335 (73.5)	1,221 (66.5)	3,419 (94.5)	1,258 (98.4)	1,185 (94.7)	976 (89.6)

**Is there accumulation of dirty clothes?**								
**Yes**	3,169 (59.3)	945 (55.8)	1,071 (58.9)	1,153 (62.8)	2,389 (66.0)	1,193 (93.3)	700 (56.0)	496 (45.5)
**No**	2,179 (40.7)	749 (44.2)	746 (41.1)	684 (37.2)	1,229 (34.0)	85 (6.7)	551 (44.0)	593 (54.5)
**Are there significant number of flies in house?**								
**Yes**	1,629 (30.5)	580 (34.2)	505 (27.8)	544 (29.6)	2,492 (65.4)	765 (59.6)	910 (71.3)	817 (65.3)
**No**	3,719 (69.5)	1,114 (65.8)	1,312 (72.2)	1,293 (70.4)	1,320 (34.6)	519 (40.4)	366 (28.7)	435 (34.7)
**Is there standing water in patio or around?**								
**Yes**	418 (7.8)	98 (5.8)	136 (7.5)	184 (10.0)	220 (5.8)	12 (0.9)	33 (2.6)	175 (14.0)
**No**	4,930 (92.2)	1,596 (94.2)	1,681 (92.5)	1,653 (90.0)	3,592 (94.2)	1,272 (99.1)	1,243 (97.4)	1,077 (86.0)

Table 7: Unadjusted and adjusted difference-in-difference estimates for primary and secondary outcomes

Variables	CMI vs. C				CM vs. C				ICC	
	Unadjusted		Adjusted		Unadjusted		Adjusted		ICC	
	DID (95% CI)	P-value	DID (95% CI)	P-value	DID (95% CI)	P-value	DID (95% CI)	P-value	BL	EL
**Primary Outcomes**										
Never immunized	-9% (-16%, -2%)	0.016	-8% (-14%, -1%)	0.020	-3% (-10%, 4%)	0.377	-2% (-8%, 4%)	0.512	0.094	0.077
Fully immunized	11% (2%, 20%)	0.018	9% (1%, 18%)	0.025	-2% (-9%, 5%)	0.547	-3% (-11%, 4%)	0.348	0.083	0.099
Sanitation Index, β (95% CI)	1.46 (0.68, 2.24)	<0.001	1.53 (0.72, 2.35)	<0.001	0.39 (-0.45, 1.24)	0.353	0.41 (-0.42, 1.24)	0.325	0.086	0.375
Drinking water index (Score 0-3), β (95% CI)	0.52 (0.25, 0.78)	<0.001	0.52 (0.25, 0.79)	<0.001	0.17 (-0.12, 0.47)	0.245	0.14 (-0.13, 0.42)	0.304	0.052	0.290
Food index (Score 0-3), β (95% CI)	0.81 (0.50, 1.12)	<0.001	0.81 (0.5, 1.13)	<0.001	0.18 (-0.21, 0.56)	0.363	0.23 (-0.15, 0.61)	0.232	0.076	0.231
Domestic household hygiene index (Score 0-6), β (95% CI)	0.14 (-0.22, 0.49)	0.442	0.20 (-0.18, 0.58)	0.290	0.04 (-0.25, 0.34)	0.768	0.04 (-0.28, 0.36)	0.812	0.058	0.436
ORS use for childhood diarrhea	31% (13%, 49%)	0.002	25% (5%, 44%)	0.014	15% (-6%, 36%)	0.165	6% (-15%, 27%)	0.545	0.043	0.066
**Secondary Outcomes**										
Open defecation	-2% (-12%, 8%)	0.727	-3% (-14%, 8%)	0.569	1% (-9%, 10%)	0.890	-1% (-10%, 9%)	0.898	0.122	0.066
Children with diarrhea during last 2 weeks	-4% (-10%, 3%)	0.240	-6% (-15%, 2%)	0.138	-1% (-7%, 6%)	0.786	-1% (-10%, 8%)	0.879	0.022	0.014
Children with ARI during last 2 weeks	-2% (-8%, 5%)	0.562	-1% (-9%, 7%)	0.870	-1% (-8%, 6%)	0.785	-3% (-11%, 4%)	0.385	0.041	0.019
Antibiotic treatment for severe cases of childhood ARI	11% (-12%, 34%)	0.331	16% (-8%, 39%)	0.191	-4% (-29%, 21%)	0.759	-1% (-29%, 28%)	0.966	0.054	0.110
Exclusive BF 6 months	24% (14%, 34%)	<0.001	25% (15%, 34%)	<0.001	11% (1%, 20%)	0.030	13% (4%, 21%)	0.007	0.028	0.047

The estimates are adjusted for child sex, age, number of siblings, mother's education, father's education, mother's occupation, father's occupation, LHW visit, wealth quintiles and clustering effect.

BL=Baseline, EL=Endline

Community Mobilization and Community Incentivization for Childhood Diarrhea and Pneumonia in Pakistan: CoMIC Trial

Cluster Randomized TrialTrial

Center of Excellence in Women & Child Health
Aga Khan University, Karachi, Pakistan

ENDLINE HOUSEHOLD SURVEY QUESTIONNAIRE

(DATA COLLECTED ON HANDHELD DEVICES)

**USERNAME**	
**PASSWORD**	

A1	Latitude (Example: 24.861462)	___________________

A2	Longitude (Example:67.009939)	___________________

Instructions for Interviewer: The GPS will record the location coordinates itself. Make sure the tablet is in open air to record location correctly.

**SECTION B: IDENTIFICATION**		
**Instruction:** This section will be asked from the head of the household or someone else at least 18 years of age or older.		
**S.NO**	**QUESTIONS**	**RESPONSES/CODES**
B1	Cluster Number	|__|__|__|__|__|__| This information will be based on the list of villages generated from the district. Each enumerator will be given a list of clusters that he/she will be visiting, and before starting the interview, this information will be entered from the same list.
B2	Cluster type	LHW covered ....1<br>LHW uncovered ....2<br>LHW partially covered 3
B3	Form Serial Number (Auto generated)	|__|__|__|__|__|__|
B4	Name of Province	Sindh 1
B5	Name of District	Tando Mohammad Khan 1
B6	Name of Tehsil/Taluka	Tando Mohammad Khan ....1<br>Tando Ghulam Hyder ....2<br>Bulri Shah Karim 3
B7	Name of Union Council	**Select from the drop down menu.**<br>1. Lakhat 2. Shaikh Bhirkio 3. Tando Saiendad 4. Dando 5. Ghulam Shah Bagrani 6. Nazarpur 7. Saeed Khan Lund 8. Mullakatiar 9. Saeed Matto 10. Saeedpur Takar
B8	Location	Urban ....1<br>Rural 2
B9	House Hold Number	|__|__|__|__|__|__|
B10	Village Name	
B11	Block/mohalla	Leave blank if Block/mohalla name is not available

**SECTION C: INTRODUCTION AND CONSENT**
**Instruction:** This section will be asked from the head of the household or someone else at least 18 years of age or older.

Assalam o Alaikum.
My name is (Name of Interviewer) and I am working with center of excellance in Women and Child Health, Aga Khan University Karachi. In collaboration with Provincial Health Department of Sindh. We are conducting an Endline HH survey on "Evaluation of a community engagement and demand creation strategy for Childhood Diarrhea and Pneumonia in Pakistan". As part of the survey, we are collecting information on the status of health of population in this project based clusters of District. The Information we are collecting relates to knowledge, attitudes and practices on child health including morbidity and mortality. This information will be used to design strategies in improving child health services by government and other healthcare providers in your area. The whole process of this interview may take approximately 30 to 40 minutes. We would very much appreciate your participation in this survey. All of the answers you provide will be confidential. Your participation in this survey is voluntary. If you do not want to provide information to a specific question or a section, please let me know. You may also withdraw from this interview at any time.

C1	At this time do you have any question for me that relates to the information I have provided you above?	Yes……………………………………………………………………………………1<br>No………………………………………………………………………………………2	
C2	May I begin the interview now?	Yes……………………………………………………………………………………1<br>No………………………………………………………………………………………2	If No, then Go to C7 and End the interview
C3	Name of the Respondent		
C4	Respondent's gender	Male……………………………………………………………………………………1<br>Female ………………………………………………… ……………………………2	
C5	Age of Respondent in completed	Years------------------Months------------------	
C6	Does your HH have a child aged 0-5-years?	Yes……………………………………………………………………………………1<br>No………………………………………………………………………………………2	
C7	Result of Household Questionnaire interview:	*Completed……………………………………………………………………………….1*<br>*No household member at home or no competent respondent at home at the time of visit…………………………………………………..…2*<br>*Entire household absent for extended period of time …………..3*<br>*Refused…………………………………………………………………………….…….4*<br>*Dwelling vacant or address not a dwelling………………………….……5*<br>*Dwelling not found ……………………………………………………….….….6*<br>*No Child between 0-5 years…………………………………………………7*<br>*Temporarily locked ………………………………………………………………..8*<br>*OTHERS (SPECIFY)---96*	

SECTION D: HOUSEHOLD MEMBERS' INFORMATION

Now I would like to ask you questions on household member information who live with you in this house and share the same kitchen. This information will be used to assess the health care needs, and services available to you and your household members in the area.

Instruction: This section will be asked from the head of the household or someone else at least 18 years of age or older.

D1	D2	D3	D4	D5	D6	D7			D8		D9	D10	D11	D12	D16
Member Serial number	Name	Relationship to Head of HH	Sex	Name of Father	Name of Mother	Date of Birth			Age		Has {Name} ever attended a school?	Education	Occupation	Marital Status	
Auto generated , starting from 01	*Please give me the names of the persons who usually live in your household, starting with the head of the household.*	*What is the relationship of {NAME} to the Head of the household?*	*Is {NAME } male or female ?*	*If father's relation is "Not Available" in the members list then record code "NA"*	*If father's relation is "Not Available" in the members list then record code "NA"*	*Record DOB from card/certificate/memory recall.*			*If date of birth cannot be recorded then record age by recall and record only months and years.*		*Yes= 1*<br>*No= 2*<br>*If no go to D11*<br>*For children less than five years, this variable will not be visible, and an auto generated NA code will appear.*	*What is the highest class (NAME) completed?*	*For children less than five years, this variable will not be visible, and an auto generated NA code will appear*		Availability
						Day	***Month***	***Year***	***Years***	***Months***					

The interviewer will ask the HH head information first, and record.<br><br>***Codes for Q# D3. Relationship with Head of household:***<br><br>*1. Head of HH*<br>*2. Wife/Husband*<br>*3. Son/Daughter*<br>*4. Son in law/Daughter in law*<br>*5. Grand child*<br>*6. Parents*<br>*7. Parents in law*<br>*8. Brother/Sister*<br>*9. Brother in law/Sister in law*<br>*10. Niece/Nephew*<br>*11. Grand Parents*<br>*12. Aunts/Uncle*<br>*13. Adopted/Step child*<br>*14. Domestic Servant*<br>*15. Other*	***Codes for Q# D4. Sex:***<br><br>***1. Male***<br>***2. Female***<br>***3. Tarnsgender***	***Codes for Q# D10. Education:***<br><br>***Please record number of years for attending formal educational institutions. This will be for number of years completed in a school. For example, if the respondent gives MA for a family member, then the data collector will enter 16 in the response cell.***<br>*0 = less than 1 YEAR completed*<br>*1 = class 1*<br>*2 = class 2*<br>*3 = class 3*<br>*4 = class 4*<br>*5 = class 5*<br>*6 = class 6*<br>*7 = class 7*<br>*8 = class 8*<br>*9 = class 9*<br>*10 = class 10*<br>*11 = class 11*<br>*12 = class 12*<br>*13 = class 13*<br>*14 = class 14*<br>*15 = class 15*<br>*16= class 16*<br>*17 = class 17*<br>*18 = class 18*<br>*19 = class 19*<br>*20= class 20*<br>*98 = Don't Know*<br>*99= Not Applicable*	***Codes for Q# D11. Occupation:***<br><br>*1. Housewife*<br>*2. Professional /Managerial*<br>*3. Clerical/technical*<br>*4. Sales and Services*<br>*5. Skilled manual*<br>*6. Unskilled manual*<br>*7. Agriculture*<br>*8. Business*<br>*9. Student*<br>*10. Unemployed*<br>*11. Retired*<br>*12. Teacher*<br>*99. Not Applicable*	***Codes for Q# D12. Marital Status:***<br><br>*1. Married*<br>*2. Unmarried*<br>*3. Widowed*<br>*4. Divorced/Separate d*

Once the member information has been completed, an eligible woman will be selected from the list of women in the member information. The interviewer then asks the respondent (if different from the eligible woman) to call the respective woman for information on the below questions that relate to the reproductive health of the women in the household.

SECTION E: SOCIO ECONOMIC STATUS OF HOUSEHOLD			
Instructions: This section will be filled by the index Mother or head of the household or someone else at least 18 years of age or older.			
**S.NO**	**QUESTIONS**	**CODES**	**SKIP**
E1	Main material of the floor<br><br>**RECORD OBSERVATION**	Earth/sand/mud……1<br>Dung……2<br>Wood planks……3<br>Palm/bamboo……4<br>Parquet or polished wood……5<br>Vinyl or asphalt strips……6<br>Ceramic tiles……7<br>Cement ……8<br>Carpet……9<br>Bricks……10<br>Lime……11<br>Marble……12<br>Other (specify)……96	
E2	Main material of the roof<br><br>**RECORD OBSERVATION**	No Roof……1<br>Thatch/Palm Leaf…… 2<br>Sod/Grass…… 3<br>Rustic Mat…… 4<br>Palm/Bamboo…… 5<br>Wood Planks……6<br>Cardboard…… 7<br>Metal/Tin/CGI……8<br>Reinforced brick cement/RCC……9<br>Metal…… 10<br>Wood/T-Iron/Mud…… 11<br>Calamine/Cement Fiber…… 12<br>Ceramic Tiles…… 13<br>Roofing Shingles…… 14<br>Bricks ……15<br>Tent/cloth……16<br>Other (specify)……96	
E3	Main material of the walls<br><br>**RECORD OBSERVATION**	No walls…… 1<br>Cane/Palm/Trunks…… 2<br>Dirt/sand/mud …… 3<br>Stones with mud…… 4<br>Bamboo with mud……5<br>Uncovered adobe…… 6<br>Plywood……7<br>Cardboard……8<br>Reused wood……9<br>Cloth/Curtain/Tent ……10<br>Carton/Plastic……11<br>Cement…… 12<br>Stone with lime/Cement…… 13<br>Bricks…… 14<br>Cement block…… 15<br>Covered adobe…… 16<br>Wood planks/Shingles…… 17<br>Palm/Bamboo……18	

		Plywood……………………………………………………19<br>Other (specify)……………………………………………96	
E4	How many rooms in this house are used for sleeping?	Number of rooms ☐☐	
E5	Do you or someone living in this Household own this dwelling?	Own……………………………………………………… 1<br>Rent……………………………………………………… 2<br>Living without paying rent…………………………3<br>Other (specify)……………………………………………96	
E6	Is the cooking usually done in the house, in a separate building, or outdoors?	**In the house**<br>Separate room used as a kitchen……………………1<br>Elsewhere in the house…………………………………2<br>In a separate building……………………………………3<br>Outdoors (open place)…………………………………… 4<br>Other (specify)……………………………………………96	
E7	What type of fuel does your household **Mainly** use for cooking?	Electricity…………………………………………………1<br>Liquefied Petroleum Gas (LPG)…………………………2<br>Natural Gas …………………………………………………3<br>Bio-gas………………………………………………………4<br>Kerosene……………………………………………………5<br>Coal, Lignite/Natural……………………………………6<br>Charcoal……………………………………………………7<br>Wood…………………………………………………………8<br>Straw/Shrubs/Grass………………………………………9<br>Agricultural Crop Residue………………………………10<br>Animal dung…………………………………………………11<br>No food cooked in household…………………………12<br>Other (specify)……………………………………………96	
E8	Does your household have?	**YES** **NO**<br>a) Electricity 1 2<br>b) Radio 1 2<br>c) Television 1 2<br>d) Landline Telephone 1 2<br>e) Refrigerator 1 2<br>f) Air conditioner 1 2<br>g) Air cooler 1 2<br>h) Chair 1 2<br>i) Cupboard 1 2<br>j) Washing Machine 1 2<br>k) Water pump 1 2<br>l) Bed 1 2<br>m) Clock 1 2<br>n) Sofa set 1 2<br>o) Camera 1 2<br>p) Sewing Machine 1 2<br>q) Personal Computer/Laptop 1 2<br>r) Internet Connection 1 2	

E9	Does any member of this household own?	a) Watch YES NO<br>b) Mobile phone 1 2<br>c) Bicycle 1 2<br>d) Motorcycle/Scooter 1 2<br>e) Animal-Drawn Cart 1 2<br>f) Car/Truck/Bus 1 2<br>g) Tractor 1 2<br>h) Boat with motor 1 2<br>i) Boat without motor 1 2<br>j) Geysers 1 2<br>k) Electric Fans 1 2<br>l) Microwave ovens 1 2	
E10	Does respondent (respondent woman) of this household own mobile phone?	Yes……………………………………………………………………1<br>No………………………………………………………………………2	
E11	Does any member of this household own any agricultural land?	Yes……………………………………………………………………1<br>No………………………………………………………………………2<br>Don't Know ……………………………………………………98	If "No" then go to E13
E12	How many acres or kanals of agricultural land do members of this household own?	Acre………………………………………………1 ☐☐☐<br>Wesa/Kanals ………… …………………………2 ☐☐☐<br>Don't Know ……………………………………… …98	
E13	Does this household own any livestock, herds, other farm animals or poultry?	Yes……………………………………………………………………1<br>No………………………………………………………………………2	If "No" then go to E15
E14	How many of the following animals do the household own?	a) Cows/Bulls/Buffalo ☐☐<br>b) Horses/Donkeys/Mules ☐☐<br>c) Goats ☐☐<br>d) Sheep ☐☐<br>e) Chickens ☐☐<br>f) Camels ☐☐	
E15	Does any member of this household have a bank account?	Yes…………………………………………………………………1<br>No……………………………………………………………………2<br>Don't Know……………………………………………………98	
E16	What language do you usually speak in your household?	Sindhi ………………………………………………… ……1<br>Dhadki ………………………………………………… ……2<br>Punjabi ………………………………………………… ……3<br>Urdu ………………………………………………… ……4<br>Balochi ………………………………………………… ……5<br>Siraiki………………………………………………… ……6<br>Barauhi………………………………………………… ……7<br>Pashto ………………………………………………… ……8<br>Kachhi……………………………………………………………9<br>Gujrati…………………………………………………………10<br>Marwari…………………………………………………………11<br>Other ………………………………………………… ….96	
E17	What is total monthly household income of this house?	Rupees---1<br>Don't want to share------------------------------------2<br>Don't Know……………………………………………………..98	
E18	What is your family's religion?	Muslim ……………………………………………………………1<br>Christian…………………………………………………………2<br>Hindu……………………………………………………………3<br>Sikh………………………………………………………………4	

		Other Specify…………………………………………………96	
E19	Do you or your family receive any support in the form of cash or kind? (select as many as apply)	BISP ---1<br>Cash Per month (other than BISP)-----------------------2<br>Food Voucher per month ---------------------------------3<br>Student Scholarship --4<br>None--5<br>Other specify 96	If "None" then go to next section F
E20	Who provides this support? (select as many as apply)	Government --1<br>NGO---2<br>Individual---3<br>Other specify 96	

"

SECTION F: REPRODUCTIVE HEALTH, MATERNAL AND CHILD MORTALITY

Instructions: Complete this section for all married women aged 15-49 years in the household

Definition of Live Birth: *Live birth refers to the complete expulsion or extraction from its mother of a product of conception, irrespective of the duration of the pregnancy, which, after such separation, breathes or shows any other evidence of life - e.g. beating of the heart, pulsation of the umbilical cord or definite movement of voluntary muscles - whether or not the umbilical cord has been cut or the placenta is attached. Each product of such a birth is considered born alive.*

Definition of StillBirth: *Stillbirth is the birth of a baby who is born without any signs of life at or after 24 weeks of pregnancy. A baby may have died during late pregnancy (called intrauterine death). More unusually, a baby may have died during labor or birth (called intrapartum death).*

Definition of Miscarriage: Miscarriage *is a term used for a pregnancy that ends on its own, within the first 20 weeks of gestation.*

**F1**	Has {Names of MWRA in the family} ever been pregnant since her marriage?	Yes……1<br>No……2	For all MWRAs If No, then go for next MWRA
**F2**	How many times {Name} has been pregnant since her marriage?	___________number	
**F3**	What were the outcome of her total pregnancies (details of all pregnancies after marriage including live, still and miscarriage)	Born alive (Live birth)…… ☐☐<br>Born dead (Stillbirth)…… ☐☐<br>Miscarriage …… ☐☐<br>Abortions…… ☐☐	
**F4**	Is she currently pregnant?	Yes……1<br>No……2<br>Don't Know ……98	If "No" or Don't know then go to F6
**F5**	How many months is she pregnant? (Gestational age)	----------weeks	
**F6**	Has there been a death of women during pregnancy, delivery and within post-partum period (within 42 days) during the last five years at your home. *If yes then ask details for below table.*	Yes……1<br>No……2	If "No" then go to F8
**F7**	If yes, then how many women died	Number----------	

**Serial No.**	**a.** Complete name of deceased mother	**b.** Age at death			**c.** Place of death 1. Home 2. Govt. Health Facility 3. Private Health facility 4. NGO health facility 5.Enroute to hospital	**e.**Date of Death			e. Cause of death *please specify* 1. Severe Bleeding (mostly bleeding after childbirth 2. Infection/Fever (mostly after childbirth) 3. High Blood Pressure during pregnancy (pre eclampsia and eclampsia) 4. Complication during delivery 5. Unsafe abortion 6. Convulsion 96. Others-(Specify)……	
		Day	Month	Year		Day	Month	Year		
1										
2										

**F8**	During last five years, has there been a death of a child under 5 years of age in your house?	Yes……1<br>No……2	If no then go to F10
**F9**	If yes, then how many children died	Number----------	

**Serial No.**	a. Complete name of deceased child	b. Father's Name of died child	c. Gender 1.Male 2.Female	d. Age at death			**c.** Place of death 1. Home 2. Govt. Health Facility 3. Private Health facility 4. NGO health facility 5.Enroute to hospital	e.Date of Death			g. Cause of death 1. Birth before 37 weeks (Preterm) 2. Difficulty in breathing (Birth Asphyxia 3. Infection (Sepsis) 4. Pneumonia 5. Convulsion 6. Diarrhea 7. Fever 8 accidents. 96. Others-(Specify)….
				Days	Month	Years		Day	Month	Year	

**1**											
**2**											

F10	Does the LHW visit your household?	Ye……………………………1<br>No……………………………2<br>Don't know……………………98	If No or Don't Know, then go to section G
F11	If yes, how frequently does she visit your household?	Weekly……………………………1<br>Fortnightly…………………………2<br>Monthly ……………………………3<br>Need based…………………………4<br>Others-(Specify)……………………96	
F12	What kind of services does a lady health worker provides to your household?	Polio Campaign……………………1<br>Routine Immunization………………2<br>Reproductive Health ……………… ….3<br>Child Illness…………………………4<br>Growth monitoring of under 5 Child...........5<br>Education/Advice on general health care including hygiene and sanitation 6	*(Multiple responses)*

**SECTION G. CHILD HEALTH (Diarrhoea)**			
This section relates to diarrheal episodes, and care related to diarrhoea especially for children younger than five years and will be asked from Index Mother.			
Now I would like to ask you some questions related to diarrhea and Acute Respiratory Infection (ARI) in the last two weeks for children less than 5 years of age in your household.			
**S.NO**	**QUESTIONS**	**CODES**	**SKIP**
G1	Has any child less than 5 years of age had diarrhea during the last 2 weeks in your household?<br>An episode of diarrhea is defined as, "(**three or more loose stools in a day/24 hours**)"	Yes .... ……………………………1<br>No……………………………………2<br>Don't know……………………………98	If No or Don't Know then go to G 32

G2	How many children had diarrhoea in the household in the last two weeks?	Number of children ☐	Select child name from members list with most recent episode of diarrhea.	
G3	Who had the most recent episode diarrhoea in the household in the last two weeks?	Serial No. of Child ---------------------	Name of Mother ---------------------	
G4	For how many days did {NAME} has/had diarrhoea?	________ (days)<br>Still ill……………………. 1	If more than one child the next section will be completed or filled for the child with the most recent episode of diarrhea.	
G5	Was there any blood in the stool?	Yes ……. ………………………………………………………1<br>No…………………………………………………………….....2<br>Don't know…………………………………………………98		
G6	Did you seek initial care/treatment when {NAME} had diarrhea?	Yes ….. ………………………………………………………1<br>No…………………………………………………………….....2<br>Don't know………………………………………………98		If yes, then go to G 8
G7	Why did you not seek care for {NAME}'s diarrheal episode?	The problem did not require care seeking…………1<br>Had no money to pay HCP……………………………..2<br>Transport was not available ……………………………3<br>A male was not present to accompany me to the health center …………………………………………………4<br>No permission from household head/husband /mother in law………………………………………………5<br>Health facility is too far ………………………………….6<br>Health facility was closed ……………………………..7<br>Poor quality service at health facility ………………..8<br>Other(Specify)--96		*(Multiple responses)* Irrespective of any response go to G 32
G8	After how many days of illness did you seek care?	--------------------- (Day/s)		
G9	Where did you seek initial care for {NAME}'s diarrheal episode?	At home …………………………………………………………1<br>Government health facility……………………………2<br>Private health facility……………………………………3<br>NGO health facility ………………………………………4		
G10	From whom did you seek initial care for {NAME}'s diarrheal episode?	Self……………………………………………………………….1<br>LHW (Lady Health Worker)……………………………2<br>Doctor…………………………………………………………3<br>Nurse …………………………………………………………4<br>Dispenser/Compounder …………………………………5<br>Chemist/Medical Store……………………………………6<br>LHV (Lady Health Visitor)………………………………7<br>Homeopathic/Hakim ………………………………………8<br>NGO staff………………………………………………………9<br>Community Midwife ……………………………………10<br>Dai/TBA ……………………………………………………11		
G11	What treatment was given to {NAME} for diarrheal illness during the initial care?	Fluid from ORS packets or pre packed liquid ……..1<br>Recommended home fluids ……………………………2<br>Antibiotic drug………………………………………………3<br>Anti-motility drug …………………………………………4<br>Zinc supplement ……………………………………………5<br>Flagyl……………………………………………………………6<br>Intravenous fluids (IV) …………………………………7<br>Home remedy ………………………………………………8		*(Multiple responses)*

		Unknown pill/syrup…………………………………………9<br>Unknown Injection…………………………………………10	
G12	Did anyone refer you to another health facility?	Yes……………………………………………………………1<br>No……………………………………………………………2	
G13	Was the child {NAME} hospitalized for diarrhea treatment?	Yes……………………………………………………………1<br>No……………………………………………………………2	If No then go to G 25
G14	Where {NAME} was hospitalized/admitted?	Government health facility……………………………1<br>Private health facility…………………………………2<br>NGO health facility ………………………………………3	
G15	After the admission did you change the facility?	Yes……………………………………………………………1<br>No……………………………………………………………2	If No then go to G18
G16	What were the reasons for changing the facility?	Cost……………………………………………………………1<br>Unavailability of staff…………………………………2<br>Unavailability of medicines…………………………3<br>Staff attitude………………………………………………4<br>The child wasn't getting better……………………5<br>Other (Specify)-- 96	
G17	Which new facility was {NAME} hospitalized?	Government health facility……………………………1<br>Private health facility…………………………………2<br>NGO health facility ………………………………………3	
G18	Who treated {NAME} for diarrhea episode at the hospitalized/admitted facility?	Doctor…………………………………………………………1<br>LHV (Lady Health Visitor)………………………………2<br>Nurse …………………………………………………………3<br>Community Midwife ……………………………………4<br>Dispenser/Compounder …………………………………5	
G19	What treatment was given to {NAME} for diarrheal illness at the hospitalized/admitted facility?	Recommended home fluids……………………………1<br>Fluid from ORS packets or pre packed liquid ……2<br>Antibiotic drug………………………………………………3<br>Anti-motility …………………………………………………4<br>Zinc supplement ……………………………………………5<br>Flagyl……………………………………………………………6<br>Intravenous fluids (IV) …………………………………7<br>Unknown pill/syrup…………………………………………8<br>Unknown Injection…………………………………………9	*(Multiple responses)*
G20	For how long {NAME} was hospitalized for the treatment of diarrhea?	__________Hours<br>__________days<br>__________Still Hospitalized	If still hospitalized, then go G.26
G21	After discharge, were you recommended a follow up visit?	Yes……………………………………………………………1<br>No……………………………………………………………2	If No then go to G25
G22	After how many days of discharge, were you recommended for a follow up visit?	-------------------- days	
G23	Did you go for a follow-up visit?	Yes……………………………………………………………1<br>No……………………………………………………………2	If Yes then go to G25
G24	What were the reasons for not having a follow up visit?	Follow-up not due yet……………………………………1<br>The problem did not require further care seeking………2<br>Had no money to pay ……………………………………3<br>Transport was not available …………………………4	*(Multiple responses)*

		A male was not present to accompany me to the health center ....5<br>No permission from household head/husband /mother in law ....6<br>Health facility is too far 7	
G25	What was the outcome of child's disease?	Cured ....1<br>Still Ill ....2<br>Died 3	
G26	How far is the health facility, where you sought treatment of {NAME} for diarrheal episode?	Kilometers ....☐ km<br>Don't know 98	
G27	What transport was used to reach health facility?	Public transport........1<br>Chingchi/ Rickshaw ....2<br>Motor-cycle /Bike........3<br>Private Car........4<br>Taxi........5<br>Ambulance........6<br>By Cycle........7<br>On foot........8<br>None (if child was treated at home) ....9<br>Other(Specify)........96	
G28	How long does it take to reach health facility, where you sought treatment of {NAME} for diarrheal episode?	Minutes ☐ min<br>Don't know 98	
G29	How much did it cost you on transport for diarrheal treatment of {NAME}?	Rupees ☐<br>Don't know 98	
G30	How much did diarrheal treatment of {NAME} cost you? (paid for *medicine + Inpatient + consultancy)*?	Rupees ☐<br>Don't know 98	
G31	Who paid the cost of diarrhea treatment of {NAME}?	Self........1<br>Hospital........2<br>Partial........3<br>Relative/friend........4<br>Other(Specify)........96	
G32	Have you heard about ORS?	Yes........1<br>No........2	If No then go to section H
G33	Are you aware about the preparatory methods of ORS (Oral rehydration salt)?	Yes........1<br>No........2	If No then go to section H
G34	Where did you learn how to prepare Oral Rehydration Solution (ORS)?	Family/friend........1<br>LHW (Lady Health Worker)........2<br>Doctor........3<br>Nurse ....4<br>Dispenser/Compounder ....5<br>Chemist/Medical Store........6<br>LHV (Lady Health Visitor)........7<br>Homeopathic/Hakim ....8<br>NGO staff........9<br>Community Midwife ....10<br>Dai/TBA ....11<br>AKU Project based staff........12<br>Other(Specify)........96	

H. CHILD HEALTH (ACUTE RESPIRATORY INFECTION (ARI))			
This section relates to diarrheal episodes, and care related to Acute Respiratory Infection especially for children younger than five years and will be asked from Index Mother.			
Now I would like to ask you some questions related to Acute Respiratory Infection (ARI) in the last two weeks for children less than 5 years of age in your household.			
**S.NO**	**QUESTIONS**	**CODES**	**SKIP**
H1	Has any child less than five years of age had fever during last two weeks in the household?	Yes ....1<br>No...2<br>Don't know.................................98	*If no, or don't know then go to H3*
H2	How many children had fever during last two weeks in the household?	Number of children ☐	
H3	Has a child had an illness with cough that he/she breathes faster than usual with short, rapid breaths or has difficulty breathing in the past two weeks?	Yes ....1<br>No...2<br>Don't know.................................98	If No or Don't Know then go to section I
H4	How many children have had an illness with cough that he/she breathe faster than usual with short, rapid breaths or have difficulty breathing in the past two weeks?	Number of children☐	If more than one child, then next section will be filled for the child with the most recent episode of fever or overall
H5	Who had the most recent illness with cough that he/she breathe faster than usual with short, rapid breaths or have difficulty breathing in the past two weeks?	Serial No. of Child<br>---------------------	Name of Mother<br>---------------------
H6	How many days did {NAME} suffer for the symptoms of cough and fast breathing?	Number of days ☐	
H7	Did you seek care/or initial treatment for {NAME} for the symptoms of cough and fast breathing?	Yes ....1<br>No...2<br>Don't know.................................98	If Yes, then go H9
H8	Why did you not seek care or treatment for {NAME}'s symptoms of cough and fast breathing?	The problem did not require care seeking...........1<br>Had no money to pay HCP..............................2<br>Transport was not available ....3<br>A male was not present to accompany me to the health center ....4<br>No permission from household head/husband /mother in law ....5<br>Health facility is too far.................................6<br>Health facility was closed ....7<br>Poor quality service at health facility ....8<br>Other(Specify)..96	*(Multiple responses)* Irrespective of any response go to section I
H9	After how many days of illness did you seek care?	----------------days	
H10	Where did {NAME} seek initial care for the symptoms of cough and fast breathing?	At home ....1<br>Government health facility..............................2<br>Private health facility.....................................3<br>NGO health facility 4	

H11	From whom did you seek initial care for the symptoms of cough and fast breathing?	Self.....1<br>LHW (Lady Health Worker).....2<br>Doctor.....3<br>Nurse.....4<br>Dispenser/Compounder.....5<br>Chemist/Medical Store.....6<br>LHV (Lady Health Visitor).....7<br>Homeopathic/Hakim.....8<br>NGO staff.....9<br>Community Midwife.....10<br>Dai/TBA.....11	
H12	What was given to {NAME} during the initial treatment for the symptoms of cough and fast breathing?	Antibiotic Pill/syrup.....1<br>Antibiotic Injection.....2<br>Antipyretics.....3<br>Cough syrup.....4<br>Home Remedies.....5<br>Anti-malarial.....6<br>Unknown pill/syrup.....7<br>Unknown Injection.....8	*(Multiple responses)*
H13	Did anyone refer you for treatment for the symptoms of cough and fast breathing to another health facility?	Yes.....1<br>No.....2	
H14	Was {NAME} hospitalized for treatment of cough and fast breathing?	Yes.....1<br>No.....2	If No then go to H26
H15	Where {NAME} was hospitalized/admitted?	Government health facility.....1<br>Private health facility.....2<br>NGO health facility.....3	
H16	Did you change the facility?	Yes.....1<br>No.....2	If No the go to H19
H17	What were the reasons for changing the facility?	Cost.....1<br>Unavailability of staff.....2<br>Unavailability of medicines.....3<br>Staff attitude.....4<br>Other(Specify).....96	
H18	In which new facility, (Name) was hospitalized?	Government health facility.....1<br>Private health facility.....2<br>NGO health facility.....3	
H19	Who treated {NAME} for the symptoms of cough and fast breathing at the facility?	Doctor.....1<br>LHV (Lady Health Visitor).....2<br>Nurse.....3<br>Community Midwife.....4<br>Dispenser/Compounder.....5	
H20	What treatment was given to {NAME} for the symptoms of cough and fast breathing at the facility?	Antibiotic Pill/syrup.....1<br>Antibiotic Injection.....2<br>Antipyretics.....3<br>Cough syrup.....4<br>Anti-malarial.....5<br>Unknown pill/syrup.....6<br>Unknown Injection.....7	*(Multiple responses)*

H21	For how long {NAME} child was hospitalized for the treatment of cough and fast breathing?	__________Hours<br>__________days<br>__________Still Hospitalized	If still hospitalized then go H27
H22	After discharge, Were you recommended a follow up visit?	Yes……………………………………………………………………1<br>No……………………………………………………………………2	If NO or not advised then go H26
H23	After how many days of discharge were you recommended for a follow up visit?	------------------- days	
H24	Have you visited for a follow-up visit?	Yes……………………………………………………………………1<br>No……………………………………………………………………2	If yes, go to H26
H25	What were the reasons for not having a follow up visit?	Follow-up not due yet……………………………………1<br>The problem did not require further care seeking………2<br>Had no money to pay ……………………………………3<br>Transport was not available …………………………4<br>A male was not present to accompany me to the health center ……………………………………………5<br>No permission from household head/husband /mother in law ………………………………………………6<br>Health facility is too far…………………………………7	*(Multiple responses)*
H26	What was the outcome of (Name's) disease?	Cured ……………………………………………………………1<br>Still Ill ……………………………………………………………2<br>Died ………………………………………………………………3	
H27	How long does it take to reach health facility, where you sought treatment of {NAME}'s for the symptoms of cough and fast breathing?	Minutes ☐ min<br>Don't Know……………………………………………………98	
H28	How far is the health facility, where you sought treatment of {NAME}'s for the symptoms of cough and fast breathing?	Kilometers ☐ km<br>Don't Know……………………………………………………98	
H29	What transport is used to reach health care facility?	Public transport………………………………………………1<br>Chingchi/ Rickshaw …………………………………………2<br>Motor-cycle /Bike……………………………………………3<br>Private Car……………………………………………………4<br>Taxi……………………………………………………………5<br>Ambulance……………………………………………………6<br>By Cycle………………………………………………………7<br>By foot…………………………………………………………8<br>None (if child was treated at home)……………………9<br>Other(Specify)………………………………………………96	
H30	How much did it cost you on transport for treatment of {NAME} symptoms of cough and fast breathing?	Rupees ☐<br>Don't know……………………………………………………98	
H31	Was {NAME} given Oxygen at the facility?	Yes……………………………………………………………………1<br>No……………………………………………………………………2<br>Don't Know ……………………………………………………98	
H32	Did {NAME} have a chest x-ray?	Yes……………………………………………………………………1<br>No……………………………………………………………………2<br>Don't Know ……………………………………………………98	

H33	At any time during illness, did {NAME} have blood taken from his/her finger or heal for testing?	Yes……1<br>No……2<br>Don't Know ……98	
H34	How much did it cost you for {NAME}'s treatment of symptoms of cough and fast breathing?<br>*(Medicine+Inpatient+consultancy)*?	__________ rupees<br>Don't know……98	
H35	Who paid the cost of treatment of symptoms of cough and fast breathing?	Self……1<br>Hospital……2<br>Partial……3<br>Relative/friend……4<br>Other(Specify)……96	
H36	Sometimes children have severe illness and should be taken to a health facility. What types of symptoms would cause you to take a child under the age of 5 to a health facility right away?	Child is not able to drink or breastfeed……1<br>Child irritable ……2<br>Child develops a fever ……3<br>Child has fast breathing ……4<br>Child has difficulty breathing ……5<br>Child has blood in stool ……6<br>Child is vomiting ……7<br>Child has convulsions……8<br>Child is lethargic ……9<br>Child is unconscious……10<br>Child's skin appears yellow ……11<br>Other(Specify)……96	*(Multiple responses)*

**SECTION I: IMMUNIZATION**			
This Section will be filled from Index Mother for all the children below the ages of 2 years (will be auto picked from household member list.)			
This section relates to the history of immunization for the child. The immunization history of the selected child will be recorded. Preference will be given to the information from an immunization card; however, in case an immunization card is not available, then verbal history will be taken from the respondent. In case you have problems in understanding the card, please take a picture of the card and discuss with your supervisor. Please refer to the training manual for further instructions.			
**I1**	Has {NAME} ever received any vaccinations to prevent him/her from getting diseases, including vaccinations	Yes……1<br>No……2<br>Don't know……98	If yes, then go to I3

	received in a campaign or immunization day or child health day?		
I2	What are the reasons for not vaccinating your child?	Did not receive advice……1<br>Religious reasons……2<br>Not necessary……3<br>Cultural Barriers……4<br>Vaccinator didn't visit at home……5<br>Other(Specify)……96	*(Multiple responses)*<br>*Irrespective of the response. Go to section J*
I3	Is there a vaccination card for (name of the child)?	Yes, seen……1<br>Yes, not seen……2<br>No……3<br>Card is at Vaccinator……4	

			Source of information for vaccine<br>1. Vaccine Card<br>2. Reported by care taker		Place of Vaccine<br>1. Govt. Hospital<br>2. Private Hospital<br>3. Home
			Card	Recall	
A	At Birth	BCG			
B		OPV-0 (Oral Polio Vaccine dose at birth)			
C	At the age of 6 weeks	OPV-1 (Oral Polio Vaccine first dose)			
D		Rota-1(Oral Vaccine for Diarrhoea 1st dose)			
E		Penta-1 (Pentavalent-1 (DPTHepBHib)			
F		PCV-1 (Pneumococcal Conjugate Vaccine 1st dose)			
G	At the age of 10 weeks	OPV-2 (Oral Polio vaccine 2nd dose)			
H		Rota-2(Oral Vaccine for diarrhoea 2nd dose)			
I		Penta-2 (Pentavalent-2 (DPTHepBHib)			
J		PCV-2 (Pneumococcal Conjugate Vaccine 2nd dose)			
K	At the age of 14 weeks	OPV-3 (Oral Polio vaccine 3rd dose)			
L		Penta-3 (Pentavalent 3 (DPTHepBHib)			
M		PCV 3 (Pneumococcal Conjugate Vaccine 3rd dose)			
N		IPV (Injectable Polio Vaccine)			
O	At the age of 9 months	Measles-1 (Measles vaccine first dose)			
		TCV (typhoid conjugate vaccine Single Dose)			
P	At the age 15 months	Measels-2 (Measles vaccine second dose)			

SECTION J: BREAST FEEDING AND NUTRITION			
This Section will be filled from Index Mother for the children below the ages of 2**4** months (name will be auto picked from household member list.)			
Now I would like to talk about breast feeding and nutrition of the youngest child at your household			
**S.NO**	**QUESTIONS**	**CODES**	**SKIP**
J1	Name of Child *(to be selected from section D)*		
J2	What was the first thing that was given to your {NAME} right after birth?	Breast milk……1<br>Milk (other than breast milk) ……2<br>Plain water ……3<br>Honey or sugar water ……4<br>Ghee, butter ……5<br>Fruit juice ……6<br>Infant formula ……7<br>Ghuttee ……8<br>Green tea ……9<br>Gripe water ……10<br>Sugar-salt-water solution……11<br>Prescribed medicine……12<br>Other(Specify)……96	
J3	How long after birth {NAME} was breastfed?	____minutes<br>____hours<br>____days<br>Never breastfeed-------666<br>Don't Know……98	
J4	Did you discard the thick milk (colostrum) that comes out of breast soon after delivery?	Yes……1<br>No……2	If No then go to J6
J5	Why did you discard the thick (colostrum) milk that comes out of breast soon after delivery?	Colostrum is harmful for newborn ……1<br>Cultural/Religious reason ……2<br>It is dirty ……3<br>It causes diarrhea ……4<br>Newborn was unable to suck ……5<br>Mother was ill ……6<br>Newborn was ill……7<br>It is heavy ……8	*(Multiple responses)*
J6	In the first three days after birth, was {NAME} given anything to drink other than breast milk?	Yes……1<br>No……2	If No then go to J9
J7	What was given to {NAME} to drink in the first three days after birth?	Milk (other than breast milk) ……1<br>Plain water ……2<br>Honey or sugar water ……3<br>Ghee, butter ……4<br>Fruit juice ……5<br>Infant formula ……6<br>Ghuttee ……7<br>Green tea ……8<br>Other(Specify)……96	*(Multiple responses)*

J8	In the last 24 hours what was given to {NAME} to eat or drink?<br><br>*Note for DC: (This question will only be asked if the respondent has child younger than 6 months)*	Breast milk……………………………………………………1<br>Milk (other than breast milk) …………………… ……2<br>Plain water ………………………………………… ……3<br>Honey or sugar water …………………………… ……4<br>Ghee, butter ……………………………………… ……5<br>Fruit juice ………………………………………… ……6<br>Infant formula …………………………………… ……7<br>Green tea ………………………………………… ……8<br>Solids/semi solid food ……………………………………9<br>Other(Specify)…………………………………………96	
J15	*(Sometimes babies are fed breast milk in different ways, for example by spoon, cup or bottle. This can happen when the mother cannot always be with her baby. Sometimes babies are breastfed by another woman, or given breast milk form another this can happen if a mother cannot breastfeed her own baby).*<br>Did (NAME) consume breast milk in any of these ways yesterday during the day or at night?	Spoon: Yes (1) | No (2) | DK (98)<br>Cup: Yes (1) | No (2) | DK (98)<br>Bottle: Yes (1) | No (2) | DK (98)	
J16	How many times did you breastfeed your child during last 24 hours?	No of Times [__] [__]<br>Did not feeded …………………………………………………2<br>Don't know……………………………………………………98	
J17	*(Now I would like to ask you about some medicines and vitamins that are sometimes given to infants)*<br><br>Was (NAME) given any vitamin, mineral supplements or other medicines yesterday during the day or at night?	Yes…………………………………………………………………1<br>No…………………………………………………………………2<br>Don't know……………………………………………………98	
J18	Was (NAME) given [LOCAL NAME FOR ORS] yesterday during the day or at night?	Yes…………………………………………………………………1<br>No…………………………………………………………………2<br>Don't know……………………………………………………98	
J10	Did you exclusively breastfeed {NAME}?<br>*Note for DC (Exclusive breastfeeding is when a child is only fed breast milk, and not given anything else. This also includes water, or liquids for stomach pain etc)*	Yes…………………………………………………………………1<br>No…………………………………………………………………2	If No then go to J12
J11	If yes, then for how long did you exclusively breastfeed {NAME}?	_____Days<br>_____months<br>Don't know……………………………………………………98	
J12	For how many days or months did you breastfeed {NAME}?	Days……………………………………………………………☐<br>Months ………………………………………………………☐<br>Still Feeding………………………………………………………1<br>Don't know……………………………………………………98	

J19	Next I would like to ask you about some liquids that (NAME) may have had yesterday during the day or at night.<br>Did (NAME) have any (ITEM FORM LIST)?		Yes (1)	No (2)	DK (98)
A	Plain water		1	2	98
B	Infant formula?		1	2	98
C	Milk such as tinned, powdered, or fresh animal milk?		1	2	98
D	Juice or juice drinks?		1	2	98
E	Clear broth?		1	2	98
F	Yogurt		1	2	98
G	Thin porridge?		1	2	98
H	Any other liquids such as [list other water based liquids available in the local setting]?		1	2	98
I	Any other liquids?		1	2	98
J20	How many times yesterday during the day or at night did (NAME) consume above responded item from list?		Only those options will display here with yes in Q No. J19		
A	Plain water		A.TIMES [__][__]		
B	Infant formula?		B.TIMES [__][__]		
C	Milk such as tinned, powdered, or fresh animal milk?		C.TIMES [__][__]		
D	Juice or juice drinks?		D.TIMES [__][__]		
E	Clear broth?		E.TIMES [__][__]		
F	Yogurt		F.TIMES [__][__]		
G	Thin porridge?		G.TIMES [__][__]		
H	Any other liquids such as [list other water based liquids available in the local setting]?		H.TIMES [__][__]		
I	Any other liquids?		I.TIMES [__][__]		
J21	Did (NAME) drink anything from a bottle with a nipple yesterday during the day or night?	Yes.....1<br>No.....2<br>Don't know.....98	If no or don't know then go to J23		
J22	What did (name) drink from the bottle with a nipple?	Top feed (formula milk) .....1<br>Animal milk.....2<br>Expressed milk.....3<br>Other(Specify).....96	If the Response is 1, go to J23, otherwise go to J24		
J23	Who advised you to use infant formula milk?	Practitioner.....1<br>Pharmacy/Pharmacist.....2<br>Relatives.....3<br>Other(Specify).....96			
J13	At what age {NAME} was given solids/semi solids?	--------------------------days<br>--------------------------months<br>Yet not given---------------------1			
J14	What solids/semi solids were first given to your child {NAME}?	Fruit .....1<br>Vegetable .....2<br>Cereals.....3<br>Egg.....4<br>Rice.....5<br>Any kind of meat .....6<br>Biscuits .....7<br>Yogurt.....8	*(Multiple responses)*		

		Commercial baby food……………………………………9<br>Suji……………………………………………………………10<br>porridge……………………………………………………11<br>kichri…… …………………………………………………12<br>Other(Specify)…………………………………………96		
J24	How many times did (NAME) eat solid, semi-solid, or soft foods other than liquids yesterday during the day or at night?	Number of times-------------------------------------- [__][__]<br>Didn't eat ……………………………………………………2<br>Don't know………………………………………………98		
J25	Please describe everything that (NAME) ate yesterday during the day or night, whether at home or outside the home.		**Yes (1)**	**No (2)**	**DK (98)**
A	Porridge, bread, rice, noodles, or other foods made from grains		1	2	98
B	Pumpkin, carrots, squash, or sweet potatoes that are yellow or orange inside		1	2	98
C	White potatoes, white yams manioc, cassava, or any other food made from roots		1	2	98
D	Any dark green leafy vegetables		1	2	98
E	Ripe mangoes, ripe papayas		1	2	98
F	Any other fruits or vegetables		1	2	98
G	Liver, kidney, heart, or other organ meats		1	2	98
H	Any meat, such as beef, lamb, goat chicken, or duck		1	2	98
I	Eggs		1	2	98
J	Fresh or dried fish, shellfish, or seafood		1	2	98
K	Any food made from beans, peas lentils, nuts, or seeds		1	2	98
L	Cheese, yogurt, or other milk products		1	2	98
M	Any oil, fats, or butter, or foods made with any of these		1	2	98
N	Any sugary foods such as chocolates, sweets candies, pastries cakes, or biscuits		1	2	98
O	Condiments for flavor, such as chilies, spices, herbs, or fish powder		1	2	98
P	Grubs, snails, or insects		1	2	98
Q	Foods made with red palm oil, red palm nut, or red palm nut pulp sauce		1	2	98

SECTION K: WATER AND SANITATION			
Instructions: This section will be filled by the index Mother or head of the household or someone else at least 18 years of age or older.			
Now I would like to talk about water and sanitation status of HH			
**S.NO**	**QUESTIONS**	**CODES**	**SKIP**
K1	What is the main source of drinking water for members of your household?	**PIPED WATER**<br>• Piped into dwelling ....1<br>• Piped into compound, yard or plot.........2<br>• Piped to neighbor ....3<br>• Public tap / standpipe ....4<br>Filtration Plant/unit..5<br>**UNDERGROUND WATER**<br>• Tube Well, Borehole ....6<br>• Hand Pump ....7<br>**DUG WELL**<br>• Protected well ....8<br>• Unprotected well ....9<br>Rainwater collection ....10<br>Tanker-truck ....11<br>Cart with small tank / drum ....12<br>Surface water (river, stream, dam, lake, pond, canal, irrigation channel) ....13<br>Bottled water ....14<br>Filtration plant on boring...15<br>Others (Specify) 96	
K2	What is the main source of water used by your household for other purposes such as cooking and hand washing?	**PIPED WATER**<br>• Piped into dwelling ....1<br>• Piped into compound, yard or plot.........2<br>• Piped to neighbor ....3<br>• Public tap / standpipe	

		.4<br>Filtration Plant/unit...5<br>**UNDERGROUND WATER**<br>• Tube Well, Borehole ....6<br>• Hand Pump ....7<br>**DUG WELL**<br>• Protected well ....8<br>• Unprotected well ....9<br>Rainwater collection ....10<br>Tanker-truck ....11<br>Cart with small tank / drum ....12<br>Surface water (river, stream, dam, lake, pond, canal, irrigation channel) ....13<br>Bottled water ....14<br>Filtration plant on boring.......................................15<br>Others (Specify) 96	
K3	Where is that drinking water source located?	In own dwelling ....1<br>In own yard / plot ....2<br>Elsewhere 3	If response is 3 then go to K4, otherwise go to K6.
K15	Who has provided you this drinking water facility?	Self...1<br>AKU Project ....2<br>Govt (Municipality/Town Committee/UC ... .3<br>Other NGO...4<br>Other(specify)................................ 96	
K4	How long does it take to get to the water source to get water and come back?	Number of minutes ....<br>Don't Know 98	
K5	Who usually goes to this source to collect water for the household?<br><br>*Probe:*<br>*Is this person under age 15? What sex?*	Adult woman (age 15+ years) ....1<br>Adult man (age 15+ years)...............................2<br>Female child (under 15) ....3<br>Male child (under 15) ....4<br>Whoever is available 5	
K6	How does that drinking water in the household taste?	Sweet ....1<br>Brackish 2	
K7	Was the water for drinking clear or muddy at the time of collection?	Clear ....1<br>Muddy/colored...2<br>Don't Know 98	
K8	Do you do anything to the water to make it safer to drink?	Yes..1<br>No...2<br>Don't know...98	If No or Don't Know then go to K10

K9	What do you usually do to make the water safer to drink?	Boil ....1<br>Add bleach / chlorine Tablet ....2<br>Strain it through a cloth ....3<br>Use water filter (ceramic, sand, composite, etc.) ....4<br>Solar disinfection ....5<br>Let it stand and settle ....6<br>Alum (Phitkari) ....7<br>Others (Specify) 96	
K10	How do you usually store drinking water in your household?	No storage..........1<br>Water Tanks ....2<br>Bottles ....3<br>Jerry Cane ....4<br>Bucket ....5<br>Clay pot..........6<br>Others (Specify) 96	
K11	What kind of toilet facility do members of your household usually use?<br><br>*Note for DC: (If not possible to determine, ask permission to observe the facility)*	**FLUSH/ POURFLUSH**<br>• Flush to piped sewer system..........1<br>• Flush to septic tank..........2<br>• Flush to soakage pit..........3<br>• Flush to somewhere else..........4<br>• Flush to unknown place/Not sure/DK where..........5<br>**PIT LATRINE**<br>• Ventilated Improved Pit latrine (VIP)….6<br>• Pit latrine with slab..........7<br>• Pit latrine without slab/Open pit..........8<br>Composting toilet..........9<br>Bucket..........10<br>No facility, Bush, Field..........11	If response is 4 aor 11 then go to section L
K12	Do you share this facility with others who are not members of your household?	Yes..........1<br>No..........2<br>Don't know..........98	If No or Don't Know then go to section L
K13	Do you share this facility only with members of other households that you know, or is the facility open to the use of the general public?	Other household only (not public) ....1<br>Public facility 2	
K14	How many households in total use this toilet facility, including your own household?	Number of household (If less than 10) ....1<br>Ten or more household ....2<br>Don't Know 98	

K16	Who has provided you this toilet facility?	Self.....1<br>AKU Project .....2<br>Govt (Municipality/Town Committee/UC .....3<br>Other NGO.....4<br>Other(specify).....96	

SECTION L: HANDWASHING			
Instructions: This section will be filled by the index Mother or head of the household or someone else at least 18 years of age or older.			
Observation & Information			
**S.NO**	**QUESTIONS**	**CODES**	**SKIP**
L1	We would like to learn about the places that household members use to wash their hands.<br>Can you please show me where members of your household most often wash their hands?	Observed .....1<br>**Not Observed**<br>• Not in dwelling/plot/yard .....2<br>• No permission to see .....3<br>• Other(specify).....96	If Not Observed, then go to L5
L2	Observe presence of water at the place for hand washing.<br>*(Verify by checking the tap/pump, or basin, bucket, water container or similar objects for presence of water).*	Water is available.....1<br>Water is not available 2	
L3	Is soap, detergent or ash/mud/sand present at the place for hand washing?	Yes, present .....1<br>No, not present.....2<br>Not Observed.....3	If No or not observed then go to L5
L4	What was available at the place of hand washing?<br>*(Record your observation.*<br>*Select all that apply).*	Bar soap .....1<br>Detergent (Powder/Liquid/Paste).....2<br>Liquid soap .....3<br>Ash/Mud/Sand 4	*(Multiple responses)*<br>Irrespective of any response go to L8

L5	Do you have any soap or detergent or ash/mud/sand in your house for washing hands?	Yes……1<br>No……2	If No then go to L8
L6	Can you please show it to me?	Yes, Shown……1<br>No, not shown……2	If No then go to L8
L7	Record your observation.	Bar soap……1<br>Detergent (Powder/Liquid/Paste)……2<br>Liquid soap……3<br>Ash/Mud/Sand……4	*(Multiple responses)*
L8	How often you wash your hands with soap or detergent or ash/mud/sand?	**Daily**<br>After using toilet……1<br>Before eating food……2<br>After eating food……3<br>After field work……4<br>After changing dippers/cleaning baby……5<br>Before feeding baby……6<br>Before cooking……7<br>Sometimes……8<br>Never……9	*(Multiple responses)*
L9	How often you take bath?	Daily…… …. …..1<br>Once in a week……2<br>Twice in a week……3<br>Thrice in a week……4<br>Other (Specify)……96	
L10	How often you brush your teeth?	Daily…… …. …..1<br>Once in a week……2<br>Twice in a week……3<br>Thrice in a week……4<br>Other (Specify)……96	
L11	What type of brush do you usually use?	Tooth Paste……1<br>Miswak……2<br>Tooth Powder……3<br>Other (Specify)……96	
**Children's Hygiene Practices**			
	Child's Name	Drop Down	
L12	How often (Name) washes his/her hands with soap or detergent or ash/mud/sand?	Daily<br>After using toilet……1<br>Before eating food……2<br>After eating food……3<br>After any kind of domestic work……4<br>After touching animals, including family pets……5<br>After being outside (playing, gardening)……6<br>After coming back from School……7<br>After visiting or handshake with any sick member or relative……8<br>Sometimes……9<br>Never……10	

L13	How often (Name) takes bath?	Daily…………………………………………………… …. …..1<br>Once in a week……………………………………………...2<br>Twice in a week……………………………………………..3<br>Thrice in a week…………………………………………….4<br>Other (Specify) ……………………………………….….96	
L14	How often (Name) brushes his/her teeth?	Daily…………………………………………………… …. …..1<br>Once in a week……………………………………………...2<br>Twice in a week……………………………………………..3<br>Thrice in a week…………………………………………….4<br>Other (Specify) ……………………………………….….96	
L15	What type of brush do (Name) usually use?	Toothpaste…………………………………………………….1<br>Miswak………………………………………………………….2<br>Tooth Powder………………………………………………..3<br>Other (Specify) ………………………………………….…..96	

SECTION N: ANTHROPOMETRIC MEASURMENTS			
Read aloud: Now we are going to do some measurements of (CHILD NAME) height, weight, and arm. Each measurement will be done at least twice.			
**S.NO**	**QUESTIONS**	**CODES**	**SKIP**
N1	Does mother allow for anthropometric measurement of her child?	*Yes ……………………………………………………. 1*<br>*No………………………………………………………. 2*	If 2 then go to N6
N2	Height measurement of mother	|__|__|__|•|__| cm	
N3	Weight measurement of mother	|__|__|__|•|__| kg	
N4	How tall is your husband (the father of Child name) than you?	*Taller than you ---------------------------------------1*<br>*Same height as you ---------------------------------2*<br>*Shorter than you--------------------------------------3*	If 2 then go to N6
N5	Approximately how much taller/shorter is he? Please record in inches the difference indicated.	*/__/__/__/•/__/ Inches……………………….1*<br>*Don't Know --98*	
N6	Name Of <U5 Child? (*Drop-down from section D*)	--	
N7	Do you think that your (Child Name) height is appropriate for his/her age?	Height is Appropriate for his/her age…….1<br>Taller than for his/her age……………………2	
N8	Do you think that your (Child Name) weight is appropriate for his/her age?	Weight is Appropriate for his/her age……1<br>higher than for his/her age……………………2	
N9	Result of anthropometric assessment of child?	Child is agree ……………………………………..1<br>Refused…………………………………………………2	If 2 or 3 then go for next available child
N10	Height measurement #1	|__|__|__|•|__| cm	Measurer 1: ID:
N11	Height measurement #2	|__|__|__|•|__| cm	Measurer 2: ID:

N12	Do the height measurements differ by >0.6 cm?	*Yes* .... *1*<br>*No*.......................... *2*	If no (differ by <0.6 cm), go to N14
N13	Height measurement #3	|__|__|__|•|__| cm	Measurer 3:ID:
N14	Weight measurement #1	|__|__|__|•|__| kg	Measurer 1: ID:
N15	Weight measurement #2	|__|__|__|•|__| kg	Measurer 2: ID:
N16	Do the weight measurements differ by >.1 kg?	*Yes* .... *1*<br>*No*.......................... *2*	If no (differ by <0.1 kg), go to N18
N17	Weight measurement #3	|__|__|__| kg	Measurer 3:ID:
N18	Left middle upper arm circumference measurement #1	|__|__|__|•|__| cm	Measurer 1: ID:
N19	Left middle upper arm circumference measurement #2	|__|__|__|•|__| cm	Measurer 2: ID:
N20	Do the middle upper arm circumference measurements differ by <.1.3 mm	*Yes* .... *1*<br>*No*.......................... *2*	If no (differ by <.1.3 mm), then end the assesment
N21	Left middle upper arm circumference measurement #3	|__|__|__|•|__| cm	Measurer 3:ID:
