# Supplementary material for: An innovative Community Mobilisation and Community Incentivisation for child health in rural Pakistan (CoMIC): a cluster-randomised, controlled trial
Source: Lancet Glob Health. 2024 Dec 18;13(1):e121–33. doi: 10.1016/S2214-109X(24)00428-5 (PMC11659842; doi:10.1016/S2214-109X(24)00428-5)
Supplement: Supplementary appendix 4 — Equitable Partnership Declaration [file mmc4.pdf]

THE LANCET
Global Health

Supplementary appendix 4

This Equitable Partnership Declaration (EPD) was submitted by the authors, and we reproduce it as supplied. It has not been peer reviewed. *The Lancet*'s editorial processes have not been applied to the EPD.

Supplement to: Das JK, Salam RA, Padhani ZA, et al. An innovative Community Mobilisation and Community Incentivisation for child health in rural Pakistan (CoMIC): a cluster-randomised, controlled trial. *Lancet Glob Health* 2025; **13:** e121–33.

Equitable Partnership Declaration questions

Researcher considerations

1. Please detail the involvement that researchers who are based in the region(s) of study had during a) study design; b) clinical study processes, such as processing blood samples, prescribing medication, or patient recruitment; c) data interpretation; and d) manuscript preparation, commenting on all aspects. If they were not involved in any of these aspects, please explain why.

   This question is intended for international partnerships; if all your authors are based in the area of study, this question is not applicable.

   This should include a thorough description of their leadership role(s) in the study. Are local researchers named in the author list or the acknowledgements, or are they not mentioned at all (and, if not, why)? Please also describe the involvement of early career researchers based in the location of the study. Some of this information might be repeated from the Contributors section in the manuscript. Note: we adhere to [ICMJE authorship criteria]() when deciding who should be named on a paper.

All the researchers (except one) were based in the region of the study during the study period.

**a) Study design:**<br>The study was conceptualised and designed by the researchers based in the region including JKD & ZAB; Funding acquisition: JKD & ZAB; Project administration: JKD, ZAP, MM, MKJ, IS, IA, SK, KM, RB, AN; Supervision: JKD;
**b) Clinical study processes:**<br>The study processes including designing, data collection, data curation and analysis was also conducted by the researchers based in the region including JKD, RAS, AR, IA, ZAP, MM, MKJ, IS, SK, KM, RB, AN, FS, SM.<br>All the study processes were supervised by JKD.
**c) Data interpretation:**<br>Formal analysis and data interpretation also involved local researchers including AR, IA, FS, A.R, SM, SC, RAS, JKD
**d) Manuscript preparation:**<br>Manuscript was initially drafted by JKD, RAS, ZAP; and then all the authors contributed to the final draft.

2. Were the data used in your study collected by authors named on the paper, or have they been extracted from a source such as a national survey? ie, is this a secondary analysis of data that were not collected by the authors of this paper. If the authors of this paper were not involved in data collection, how were data interpreted with sufficient contextual knowledge?

   The Lancet Global Health *believe contextual understanding is crucial for informed data analysis and interpretation.*

The data for the CoMIC trial was collected and analysed by the authors named on the paper.

3. How was funding used to remunerate and enhance the skills of researchers and institutions based in the area(s) of study? And how was funding used to improve research infrastructure in the area of study?

   Potentially effective investments into long-term skills and opportunities within institutions could include training or mentorship in analytical techniques and manuscript writing, opportunities to lead all or specific aspects of the study, financial remuneration rather than requiring volunteers, and other professional development and educational opportunities.

   Improvements to research infrastructure could be funding of extended trial designs (such as platform trials) and use of master protocols to enable these designs, establishment of long-term contracts for research staff, building research facilities, and local control of funding allocation.

**Skills:** The funding was utilised to enhance training and provide mentorship to the junior researchers involved in the CoMIC trial for designing and implementing future large scale community-based trials and conducting data analysis.
**Research infrastructure:** The funding for the CoMIC trial was also utilised to build research infrastructure for large data collection and data analysis.

4. How did you safeguard the researchers who implemented the study?

   Please describe how you guaranteed safe working conditions for study staff, including provision of appropriate personal protective equipment, protection from violence, and prevention of overworking.

Staff safety was ensured, and adequate working conditions were maintained through the trial period. A temporary research office was set-up in the study area after seeking all regulatory approvals to ensure staff safety. The office was located within the study district and included office space as well all the basic amenities including well-equipped kitchen and separate toilet facilities for men and women for cultural appropriation. Throughout the study period, cleaning staff was also hired to maintain the cleanliness at the office premises on a day-to-day basis. Transport was also arranged to pick and drop the data collectors from the research office to the fields on a daily basis to ensure their safety. The staff was safeguarded and provided all privileges according to the Aga Khan University protocols.

Benefits to the communities and regions of study

5. How does the study address the research and policy priorities of its location?

   How were the local priorities determined and then used to inform the research question? Who decided which priorities to take forward? Which elements of the study address those priorities?

CoMIC trial was unique in the sense that it began with a distinctly planned formative phase to conduct need assessment and priority setting for the study population. This assisted in identifying the existing community practices and barriers and facilitators for improving child health behaviours. The community identified inclusive community engagement and demand creation strategies tied to conditional short term tangible incentives could help foster behaviour change. This was followed by intervention delivery which involved non-cash incentives decided by the

community themselves based on a formal needs assessment and prioritization exercise. The common incentives included water and sanitation facilities and these incentives were then delivered by the study team and the total cost was shared by the project (75%) and the community (25%) to improve ownership and sustainability.

6. How will research products be shared in the community of study?

For instance, will you be providing written or oral layperson summaries for non-academic information sharing? Will study data be made available to institutions in the region(s) of study? The Lancet Global Health *encourages authors to translate the summary (abstract) into relevant languages after paper editing; do you intend to translate your summary?*

The findings from the study will be shared with the community in local language through local dissemination events and we also aim to translate the abstract in local language.

7. How were individuals, communities, and environments protected from harm?

a) How did you ensure that sensitive patient data was handled safely and respectfully? Was there any potential for stigma or discrimination against participants arising from any of the procedures or outcomes of the study?

We did not anticipate any potential stigma associated with the study participation. However, the all the data sets were kept anonymous for analysis.

b) Might any of the tests be experienced as invasive or culturally insensitive?

The study did not involve any invasive or culturally sensitive interventions. However, we ensured that only female interviewers took consent and interviewed female respondents to be culturally appropriate.

c) How did you determine that work was sensitive to traditions, restrictions, and considerations of all cultural and religious groups in the study population?

We did not anticipate the intervention delivery to be sensitive, however, during the formative phase, we collected information on the major community groups (tribes/sects) residing in study villages and then collated all the information to form community groups (clusters) so that the clusters formed were uniform in terms of the tribes and sects in the study area.

d) Were biowaste and radioactive waste disposed of in accordance with local laws?

There were no biowaste and radioactive waste involved in the study.

e) Were any structures built that would have impacted members of the community or the environment (such as handwashing facilities in a public space)? If so, how did you ensure that you had appropriate community buy-in?

The community was actively engaged before the start of the study in the formative phase. The study built 500 toilets and water supply structures and these were decided by the community themselves based on a formal needs assessment and prioritization exercise. Moreover, the total cost was shared by the project as well as the community to ensure ownership and sustainability.

f) How might the study have impacted existing health-care resources (such as staff workloads, use of equipment that is typically employed elsewhere, or reallocation of public funds)?

We do not anticipate any impact on the existing health-care resources.

8. Finally, please provide the title (eg, Dr/Prof, Mr/Mrs/Ms/Mx), name, and email address of an author who can be contacted about this statement. This can be the corresponding author.

Name: Dr Jai K Das
Email: jai.das@aku.edu
